# Acrylamide in food: Progress in and prospects for genetic and agronomic solutions

**DOI:** 10.1111/aab.12536

**Published:** 2019-08-07

**Authors:** Sarah Raffan, Nigel G. Halford

**Affiliations:** ^1^ Plant Sciences Department Rothamsted Research Harpenden UK

**Keywords:** cereals, crop composition, crop disease, crop nutrition, crop storage, food safety, potato, processing contaminants, rye, wheat

## Abstract

Acrylamide is a processing contaminant and Group 2a carcinogen that was discovered in foodstuffs in 2002. Its presence in a range of popular foods has become one of the most difficult problems facing the food industry and its supply chain. Wheat, rye and potato products are major sources of dietary acrylamide, with biscuits, breakfast cereals, bread (particularly toasted), crispbread, batter, cakes, pies, French fries, crisps and snack products all affected. Here we briefly review the history of the issue, detection methods, the levels of acrylamide in popular foods and the risk that dietary acrylamide poses to human health. The pathways for acrylamide formation from free (non‐protein) asparagine are described, including the role of reducing sugars such as glucose, fructose and maltose and the Maillard reaction. The evolving regulatory situation in the European Union and elsewhere is discussed, noting that food businesses and their suppliers must plan to comply not only with current regulations but with possible future regulatory scenarios. The main focus of the review is on the genetic and agronomic approaches being developed to reduce the acrylamide‐forming potential of potatoes and cereals and these are described in detail, including variety selection, plant breeding, biotechnology and crop management. Obvious targets for genetic interventions include asparagine synthetase genes, and the asparagine synthetase gene families of different crop species are compared. Current knowledge on crop management best practice is described, including maintaining optimum storage conditions for potatoes and ensuring sulphur sufficiency and disease control for wheat.

## Graphical abstract

The presence of acrylamide in a range of popular foods has become one of the most difficult problems facing the food industry and its supply chain. Wheat, rye and potato products are major sources of dietary acrylamide, with biscuits, breakfast cereals, bread (particularly toasted), crisp bread, batter, cakes, pies, French fries, crisps and snack products all affected. This review covers all aspects of the problem, with its main focus on the genetic and agronomic approaches being developed to reduce the acrylamide‐forming potential of potatoes and cereals, including variety selection, plant breeding, biotechnology and crop management.

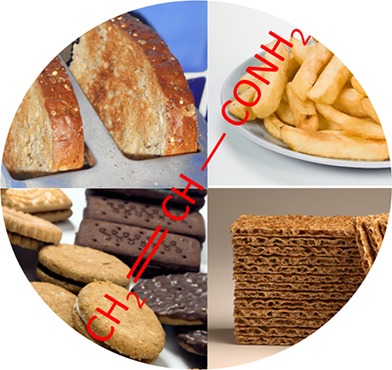

## 1 INTRODUCTION

**Figure 1:**
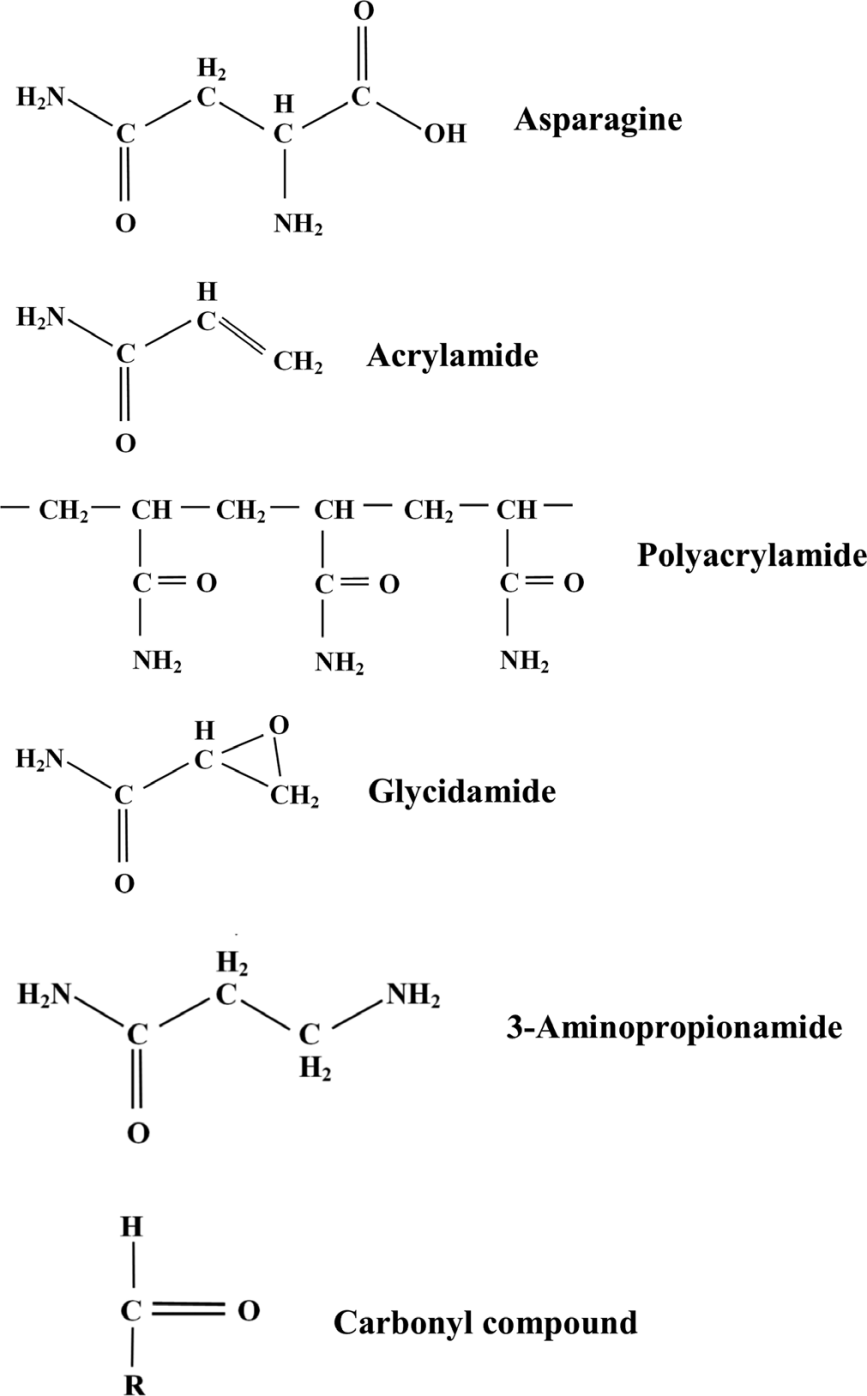
Diagram showing the structures of, from top to bottom, asparagine (C_4_H_8_N_2_O_3_), acrylamide (C_3_H_5_NO), acrylamide chains in polyacrylamide, glycidamide (C_3_H_5_NO_2_) (a metabolite of acrylamide), 3‐aminopropionamide (C_3_H_8_N_2_O) (an intermediate in one of the pathways proposed for acrylamide formation), and a carbonyl compound

Acrylamide (C_3_H_5_NO) (Figure 1) is a white, odourless, crystalline, water‐soluble solid. As a monomer, it is classified as an extremely hazardous substance in the United States and a serious health hazard with acute toxicity in the European Union. Acrylamide is a potent neurotoxin, affects male reproduction, causes birth defects and is carcinogenic in laboratory animals (reviewed by Friedman (2003); CONTAM Panel (European Food Safety Authority Panel on Contaminants in the Food Chain) (2015)) and classed as a Group 2A carcinogen (probably carcinogenic to humans) by the International Agency for Research on Cancer (IARC), the specialised cancer agency of the World Health Organization (IARC, 1994). It is readily absorbed through the skin, by inhalation and from the gastro‐intestinal tract. Once absorbed, it spreads rapidly around the body and is metabolised to produce glycidamide (C_3_H_5_NO_2_) (Figure 1). Glycidamide is also toxic and may actually be responsible for the genotoxic and carcinogenic effects attributed to acrylamide because it forms adducts with DNA both in vitro and in vivo in human and animal tissues, whereas acrylamide‐DNA adducts have only been observed in vitro (reviewed by CONTAM Panel (2015)).

Acrylamide forms a polymer, polyacrylamide (Figure 1), which is not considered to be toxic. Indeed, polyacrylamide is used as a flocculant in wastewater and sewage treatment, and has a variety of other industrial uses, as well as being a familiar laboratory chemical. Although polyacrylamide is considered safe, it may contain a small concentration of monomeric acrylamide as an impurity, and monomeric acrylamide is, therefore, a potential water pollutant, with a guideline value for its presence in drinking water set by the World Health Organisation at 0.5 μg per litre, or parts per billion. In reality, it would be extremely difficult to detect acrylamide at that concentration, and if acrylamide is detectable at all the water is considered to be polluted.

Acrylamide is also present in tobacco smoke, and studies on smokers and people who have suffered occupational exposure as a result of its use in industrial processes are the main sources of information on the toxicity of acrylamide to humans. There are several possible quantitative markers for exposure, but those most commonly used arise from reactions of acrylamide and glycidamide with proteins, notably haemoglobin in the blood. Acrylamide reacts with the N‐terminal valine residues of globin chains to produce the adduct N‐(2‐carbamoylethyl)valine (CEV), and the ratio of adducts to globin chains provides the measure of acrylamide exposure. The adducts are removed from the N‐terminal end of the protein by N‐alkyl Edman degradation, a modification of the Edman degradation method used in amino acid sequence analysis, and detected using mass spectrometry. In 1993, a study of workers involved in acrylamide manufacture found elevated levels of these adducts and correlated adduct levels with evidence of peripheral neuropathy (Bergmark, Calleman, He, & Costa, 1993).

Emma Bergmark, who was a member of the team that performed that study, subsequently showed that CEV adducts were detectable in laboratory workers who were using polyacrylamide gels for electrophoresis (PAGE), at higher levels than in non‐smoking controls (Bergmark, 1997). Importantly in the context of this review, Bergmark also noted higher than expected levels of adducts in those non‐smoking controls and remarked that “the origin of this background is not known.” Other studies reported high levels of adducts in control groups, and this remained unexplained until 2000, when it was reported that acrylamide formed during the frying of animal feed and was associated with the formation of CEV adducts in rats fed the fried feed (Tareke, Rydberg, Karlsson, Eriksson, & Törnqvist, 2000). It was concluded that cooked food was probably a major source of acrylamide exposure. However, it was a subsequent study of acrylamide formation in common cooked foods, including commercial potato products and crispbreads, that really alerted the world to the fact that acrylamide was present in the human diet (Tareke, Rydberg, Karlsson, Eriksson, & Törnqvist, 2002). Subsequent studies found no acrylamide in uncooked food, and acrylamide can therefore be classified as a processing contaminant. We define processing contaminants as substances that are produced in a food when it is cooked or processed, are not present or are present at much lower concentrations in the raw, unprocessed food, and are undesirable either because they have adverse effects on product quality or because they are potentially harmful (Curtis, Postles, & Halford, 2014). The study also found that acrylamide did not form to detectable levels in any of the boiled foods that were analysed, and acrylamide continues to be associated predominantly with fried, baked, roasted or toasted foods. The publication of this study (Tareke et al., 2002) led to widespread concern in the food industry, its suppliers and regulatory authorities over the presence of acrylamide in food, and 2002 is generally regarded as the starting point for efforts made to address the problem.

## 2 DETECTION

The amount of acrylamide present in a food sample can be measured using either gas or liquid chromatography coupled with mass spectrometry (GC–MS or LC–MS/MS). In the GC–MS method, the acrylamide has to be brominated to produce 2,3‐dibromopropionamide. This is converted to 2‐bromopropenamide by dehydrobromination with triethylamine, and 2‐bromopropenamide is the compound that is actually loaded into the GC–MS. This makes the GC–MS method considerably more laborious than the LC–MS/MS method and the LC–MS/MS method has become more popular in the last decade or so as the equipment has become more widely available. However, the GC–MS method continues to be used in some laboratories because the equipment is cheaper, and the Comité Européen de Normalisation (European Committee for Standardisation) (CEN) has developed standard methods for both (EN 16618:2015 and FprCEN/TS 17083).

## 3 LEVELS OF ACRYLAMIDE IN POPULAR FOODS

**Figure 2:**
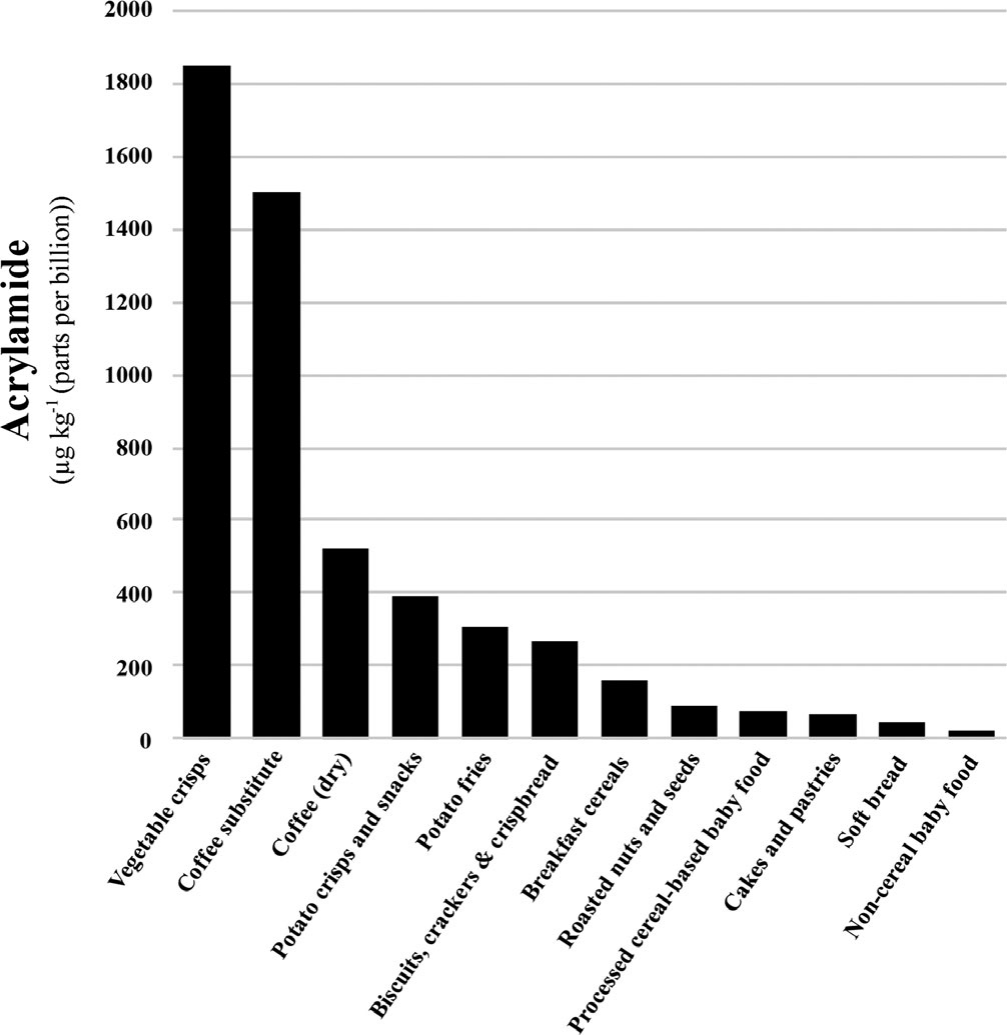
Mean acrylamide levels in selected food groups or products. Data from EFSA Panel on Contaminants in the Food Chain (CONTAM Panel, 2015)

Data on the presence of acrylamide in food in Europe has been collected since 2003, first by the European Commission Joint Research Centre's Institute for Reference Materials and Measurements and since 2007 by Member States, who have supplied data to be compiled by the European Food Safety Authority (EFSA). The data has been analysed and published in a series of reports (CONTAM Panel, 2015; EFSA, 2009, 2010, 2011, 2012). These reports have been extremely important because they informed the development of what the Commission calls its risk management measures for acrylamide (see section below on the evolving regulatory situation). The data from the 2015 CONTAM Panel report (CONTAM Panel, 2015) included data supplied by six food associations as well as Member States, and is shown graphically for a selection of foods in Figure 2. The highest levels of acrylamide were detected in vegetable crisps and coffee substitutes: the mean for chicory‐based coffee substitute was 2,942 μg kg^−1^, while its 95th percentile figure was 4,500 μg kg^−1^. Dry coffee, which is consumed in much higher quantities, had a mean of 522 μg kg^−1^. Other popular foods, such as potato crisps, potato fries, breakfast cereals, biscuits and crispbreads, all had mean values in the hundreds of μg kg^−1^. These data explain why the discovery of acrylamide in food was such a shock for everyone with a stake in the food supply chain and its regulation: the levels of acrylamide found in popular foods far exceed the tolerance level set for drinking water, and a wide range of staple foods is affected.

**Figure 3:**
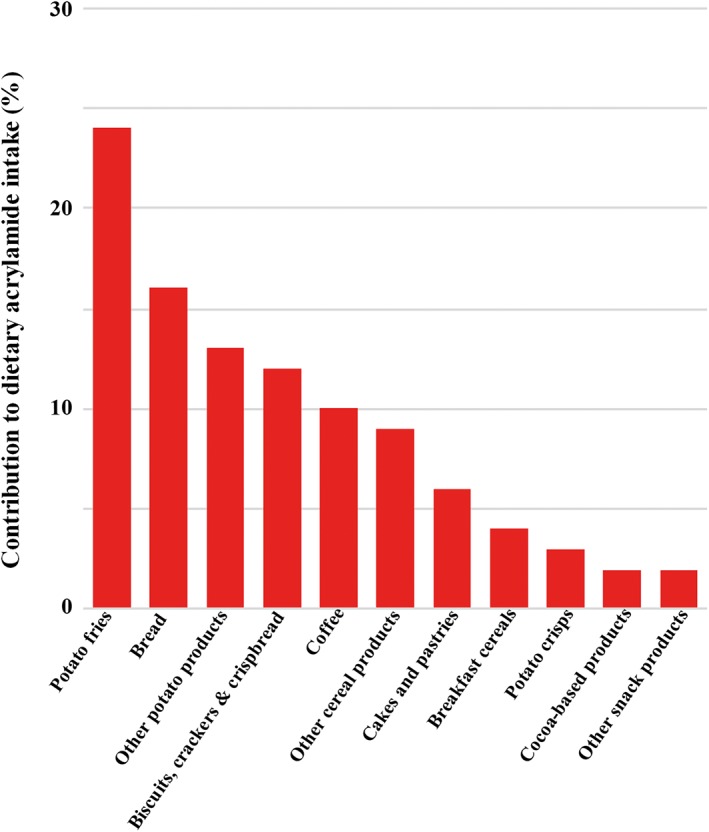
Graphical representation of the contributions of different food types to dietary acrylamide intake in the European Union, derived from the representation of multiple datasets in the EFSA Panel on Contaminants in the Food Chain report of 2015 (CONTAM Panel, 2015)

The 2011 and 2015 reports (CONTAM Panel, 2015; EFSA, 2011) also included estimates of the contribution of different foods to dietary intake in different European Union Member States. The CONTAM Panel report did not give definitive numbers to this, but summarised the data from 16 studies in the form of a table giving the number of studies that had placed the contribution of each food type with a range of 0–5%, 5–10%, 10–25%, 25–50% and > 50%. We have estimated the contribution of each food type for adults from those data, and represented the results graphically in Figure 3. In comparing Figures 2 and 3, it is striking that the two food types shown in Figure 2 with the highest levels of acrylamide, vegetable crisps and coffee substitute, do not even feature as separate categories in Figure 3. This is because the contribution of a food type to dietary intake depends on its levels of consumption as well as its acrylamide content. Bread, on the other hand, while towards the bottom end of acrylamide levels in Figure 2, is the second highest contributor to intake because so much of it is consumed and because a significant proportion of it is toasted before consumption, which increases the levels of acrylamide considerably.

The actions that the food industry and regulators have taken imply that their attention has been focussed mainly on the sort of data represented in Figure 2. Hence, breakfast cereals have received more attention than bread, for example, and potato crisps have received more attention than perhaps their contribution to total dietary intake warrants. However, arguably the data shown in Figure 3 are at least as important as the data in Figure 2, if not more so, and reducing the levels of acrylamide in bread would have much more impact on acrylamide intake than reducing the levels in breakfast cereals or potato crisps.

## 4 RISK REPRESENTED BY DIETARY ACRYLAMIDE INTAKE

The United Nations' Food and Agriculture Organisation (FAO) and World Health Organisation (WHO) Joint Expert Committee on Food Additives (JECFA) issued an opinion on the risks posed by dietary acrylamide in 2006 (JECFA, 2006). It stated that while adverse neurological effects were unlikely at the estimated average dietary exposure, morphological changes in nerves could not be excluded for individuals with high exposure. In addition, for a compound that was both genotoxic and carcinogenic, the margins of exposure indicated a health concern. A margin of exposure is defined as the ratio of the level at which a small but measurable effect is observed to the estimated exposure dose, or, alternatively, as the ratio of the maximum no‐observed‐adverse‐effect‐level to the estimated exposure dose. JECFA issued a second opinion in 2011, making almost identical statements, and similar conclusions were drawn by the EFSA CONTAM Panel for their report in 2015 (CONTAM Panel, 2015).

JECFA calculated the average dietary acrylamide exposure for the general population to be 1 μg kg^−1^ bodyweight per day, with consumers in the high percentile (i.e., those eating the most acrylamide‐containing foods) exposed to 4 μg kg^−1^ bodyweight per day. The CONTAM Panel produced similar figures, estimating the mean exposure to be between 0.4 and 1.9 μg kg^−1^ body weight per day, across all age groups, with the 95th percentile at 0.6 to 3.4 μg kg^−1^ body weight per day. These exposure figures are much lower than the doses used in rodent toxicology tests. Estimating the risk of dietary acrylamide intake from rodent toxicology data, therefore, involves extrapolation from effects of high doses over a relatively short period of time to much lower doses over a much longer period, as well as from a rodent to a human system. Data from occupational exposure studies, such as those described above, also typically involve higher levels of acrylamide than are acquired in the diet, as well as a relatively small number of people. The only other sources of information are epidemiological studies.

After assessing all of the evidence, the CONTAM Panel (2015) concluded that the level of exposure to acrylamide from the diet was insufficient to cause neurological, reproductive or developmental effects. It also considered that the results of epidemiological studies had been too inconsistent to enable firm conclusions to be drawn on links between dietary acrylamide exposure and cancer, and that its opinion on the risk of dietary acrylamide causing cancer had to be based primarily on the results of animal toxicology studies. The Panel, therefore, came to a similar conclusion to the one arrived at by JECFA 10 years earlier, which was that the margins of exposure for acrylamide indicated “a concern for neoplastic effects” (CONTAM, 2015). Although this conclusion did not depart from the position adopted by JECFA, the CONTAM report appears to have forced the hand of the European Commission to strengthen what it calls its risk management measures.

Very recently, a unique mutational “signature” has been linked to acrylamide and its metabolite, glycidamide (Zhivagui et al., 2019). This signature was found in approximately one third of the 1,600 tumour genomes analysed in the study, derived from 19 human tumour types from 14 organs. Of these, 184 liver tumour samples and 217 tumours of 15 other cancer types were found to carry this mutation but not mutations that are associated with the action of benzo[a]pyrene, another major mutagen in tobacco smoke. The mutations in these samples were therefore considered likely to reflect exposure to acrylamide that was not related to tobacco smoking; in other words, dietary or occupational exposure. The paper reporting the study presented no evidence to show that the mutations found in the tumour genomes had caused the tumours to develop. Nevertheless, the title of the paper claimed that it had revealed the “widespread contribution of acrylamide exposure to carcinogenesis in humans,” and given that the study was performed at highly respected laboratories, including those of the IARC, it is likely to prompt further regulatory action.

## 5 ACRYLAMIDE FORMATION

Shortly after the discovery of acrylamide in popular foods (Tareke et al., 2002), it was shown that acrylamide could form from free (soluble, non‐protein) asparagine (Figure 1) and reducing sugars within the Maillard reaction (Mottram, Wedzicha, & Dodson, 2002; Stadler et al., 2002). Both free asparagine and reducing sugars are, therefore, widely referred to as the precursors for acrylamide formation, although the carbon skeleton of the acrylamide that forms is derived entirely from asparagine (Figure 1). The most abundant reducing sugars in crop plants are glucose, fructose and maltose.

The Maillard reaction takes its name from the French chemist, Louis Camille Maillard, who first described it in 1912 (Maillard, 1912), although the steps in the reaction as they are understood today were first proposed by an American chemist, John Hodge, in 1953, as the Hodge Scheme (Hodge, 1953). The Maillard reaction as Hodge described it comprises a series of non‐enzymatic reactions between sugars and amino groups, promoted by high temperature (>120°C) and low moisture content, hence its occurrence mainly in cooked foods prepared by frying, baking, roasting and toasting. The products of the Maillard reaction include melanoidin pigments and complex mixtures of compounds that impart flavour and aroma, including heterocyclic compounds such as pyrazines, pyrroles, furans, oxazoles, thiazoles and thiophenes (Halford, Curtis, Muttucumaru, Postles, & Mottram, 2011; Mottram, 2007). The particular compounds that are formed give different cooked foods their signature flavour and aroma, which means that any measures taken to reduce acrylamide formation are also likely to affect the characteristics that define product types and distinguish one brand from another, making the problem all the more intractable.

The Maillard reaction is complex and multi‐step, and we will not describe it in detail here. It is initiated by the condensation of the carbonyl group of a reducing sugar with the amino group of an amino acid or other amino compound, producing a Schiff base (a type of imine; that is, a compound containing a carbon‐nitrogen double bond, in the case of a Schiff base with the nitrogen atom attached to an organic group). Cyclisation and acid‐catalysed rearrangement gives rise to Amadori rearrangement products from glucose and maltose and Heyns rearrangement products from fructose, and these undergo enolisation, deamination, dehydration and fragmentation to give rise to sugar dehydration and fragmentation products containing one or more carbonyl groups (Figure 1), including deoxyosones, heterocyclic furfurals, furanones, pyranones, dicarbonyls (C2‐C3) and hydroxycarbonyls. These carbonyl compounds may contribute to flavour characteristics in their own right but are highly reactive and can undergo further reactions with free amino acids and other amines. One of these reactions is Strecker degradation, involving the deamination and decarboxylation of an amino acid to give an aldehyde, an α‐aminoketone and carbon dioxide, and it is a Strecker‐type degradation of asparagine that produces acrylamide (Zyzak et al., 2003). The asparagine reacts with Maillard reaction‐derived dicarbonyl or hydroxycarbonyl compounds to produce a Schiff base. This can be converted to acrylamide by decarboxylation followed by the removal of a substituted imine, or it can be converted to 3‐aminopropionamide (Figure 1) by the elimination of a carbonyl group, and the 3‐aminopropionamide converted to acrylamide by the removal of ammonia (Granvogl & Schieberle, 2006; Granvogl, Wieser, Koehler, Von Tucher, & Schieberle, 2007).

**Figure 4:**
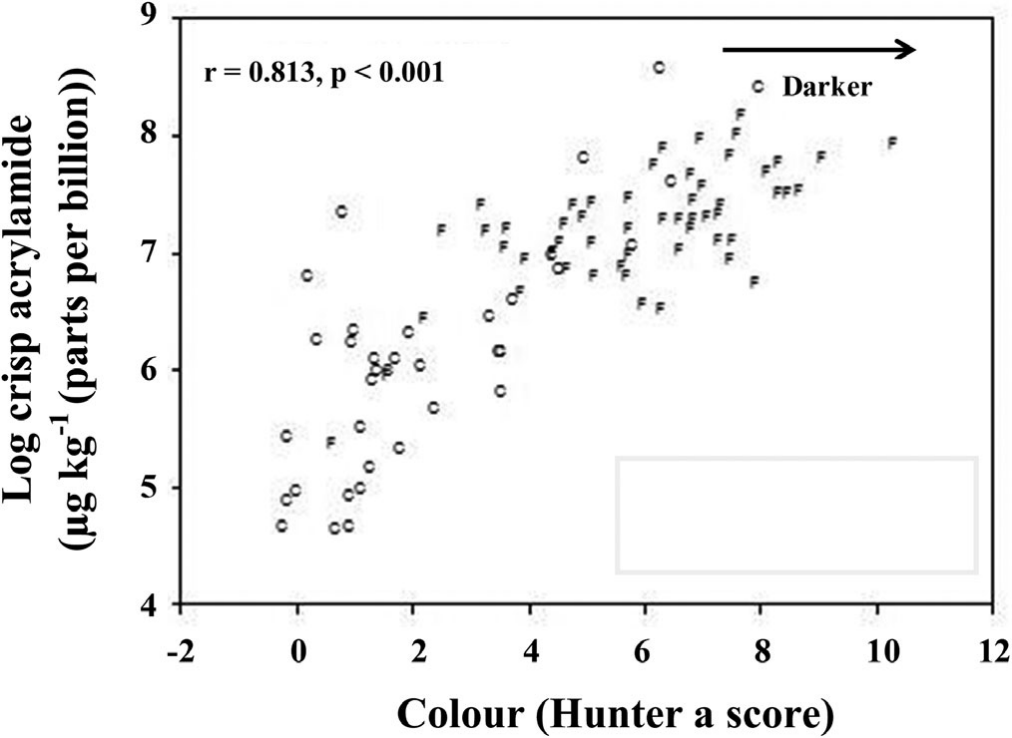
Relationship between acrylamide formation and colour (on the Hunter a scale) in potato crisps. Replotted from data published by Halford et al. (2012). Data points are shown for crisping (C) and French fry (F) potato types

The fact that colour forms via similar pathways to acrylamide means that there is usually a strong correlation between acrylamide formation and colour (Figure 4). The downside to this is that measures taken to reduce acrylamide formation are likely to affect product colour as well. The upside is that colour can be used as an indicator of acrylamide formation and has become an important quality control parameter. It was also the basis for the UK Food Standards Agency “Go for Gold” campaign, which was launched in 2017 with the aim of persuading consumers and restaurant/catering businesses to fry, bake, roast and toast food to a light brown colour (Food Standards Agency, 2017).

Alternative sources that have been proposed for acrylamide in food include its pyrolytic formation in heated gluten (Claus, Weisz, Schieber, & Carle, 2006), but the mechanism by which acrylamide could form from protein is not known. However, dried fruit, such as prunes and dates, have been shown to contain some acrylamide (CONTAM Panel, 2015) and while elevated temperatures are used in drying systems these rarely exceed 60°C. Unless the Maillard reaction can proceed at 60°C when moisture levels are low, other mechanisms must be responsible.

## 6 THE EVOLVING REGULATORY SITUATION

As we have already stated, from 2003–2006, data on acrylamide levels in food in the EU were compiled by the Commission Joint Research Centre's Institute for Reference Materials and Measurements. In 2005, EFSA's Scientific Panel on Contaminants in the Food Chain (CONTAM) requested that data on acrylamide levels in food in the European Union should be collected by European Union Member States and reported to EFSA. The aim of this was to enable an assessment to be made of the success of measures taken by the food industry to reduce the presence of acrylamide in its products, which had been compiled by FoodDrinkEurope (then known as the Confédération des Industries Agro‐Alimentaires de l'UE [CIAA]) into an “Acrylamide Toolbox” (FoodDrinkEurope, 2019). The implication was that the Commission would strengthen its risk management measures on acrylamide in food if industry measures were seen to be ineffective. Hence the European Union's regulations on the presence of acrylamide in food have been guided by a series of EFSA reports arising from analyses of these data. In 2007, the European Commission issued Recommendation 2007/331/EC on the monitoring of acrylamide levels in food in 2007, 2008 and 2009, and EFSA reported on the 2007 data and data from previous years in 2009 (EFSA, 2009), concluding that there was “no consistent trend across food groups towards lower levels of acrylamide.”

In 2010, Commission Recommendation 2010/307/EU (European Commission, 2010) required Member States to continue to collect data on acrylamide levels in food until further notice. A second EFSA report (EFSA, 2010) covering the period up to 2008 concluded that a trend towards lower levels of acrylamide was becoming apparent but that it was limited to certain food groups, with levels in most food groups remaining unchanged and, in a few cases, showing a rise. This led to Commission Regulation C (2010) 9681 final (European Commission, 2011), which introduced the concept of Indicative Values for acrylamide in food, with the competent authorities in Member States required to investigate if the acrylamide levels in a food were found to exceed the Indicative Value and ensure that the manufacturer addressed the problem. The Indicative Values that were set for some popular foods are given in Table 1. Indicative Values were not meant to be regulatory limits or safety thresholds; rather, they were set at levels that the Commission calculated the food industry ought to be able to achieve, based on EFSA's monitoring data, with different food categories being assigned different Indicative Values. However, they were often misinterpreted as safety thresholds by the media.

**Table 1:** Indicative values and benchmark levels for acrylamide in food, set by the European Commission

Food	Indicative value 2011 (ppb)	Indicative value 2013 (ppb)	Benchmark level 2017 (ppb)
French fries	600	600	500
Potato crisps	1,000	1,000	750
Soft bread (wheat)	150	80	50
Soft bread (other)		150	100
Breakfast cereals: bran products, whole grain cereals, gun puffed grain	400	400	300
Breakfast cereals: wheat and rye based		300	300
Breakfast cereals: maize, oat, spelt, barley and rice based		200	150
Biscuits	500	500	350
Crackers	500	500	400
Crispbread	500	450	350
Gingerbread	—	1,000	800
Cereal‐based baby foods	100	50	40
Baby foods (not cereal based) without prunes	80	50	
Baby foods (not cereal based) with prunes		80	
Biscuits and rusks for infants and young children	250	200	150
Roast coffee	450	450	400
Instant coffee	900	900	850
Coffee substitute (cereal‐based)	—	2,000	500
Coffee substitute (chicory)	—	4,000	4,000

Further EFSA reports followed in 2011 and 2012 (EFSA, 2011, 2012). Importantly, these found little change in acrylamide levels over the monitoring period, with some product categories showing a small decrease but others, including crispbread, French fries from fresh potatoes and instant coffee actually showing an increase, and between 3% and 30% of samples in different food categories exceeding the Indicative Value set for that product type. The Commission's response was Recommendation 2013/647/EU (European Commission, 2013), which revised and, in many cases, reduced Indicative Values (Table 1). This was followed in 2015 by the publication of EFSA's assessment of the risk posed by dietary acrylamide, prepared by its CONTAM Panel (2015). The European Commission then embarked on another process of strengthening its risk management regulations for acrylamide, and Commission Regulation (EU) 2017/2158 (European Commission, 2017) came into force on April 11, 2018.

**Figure 5:**
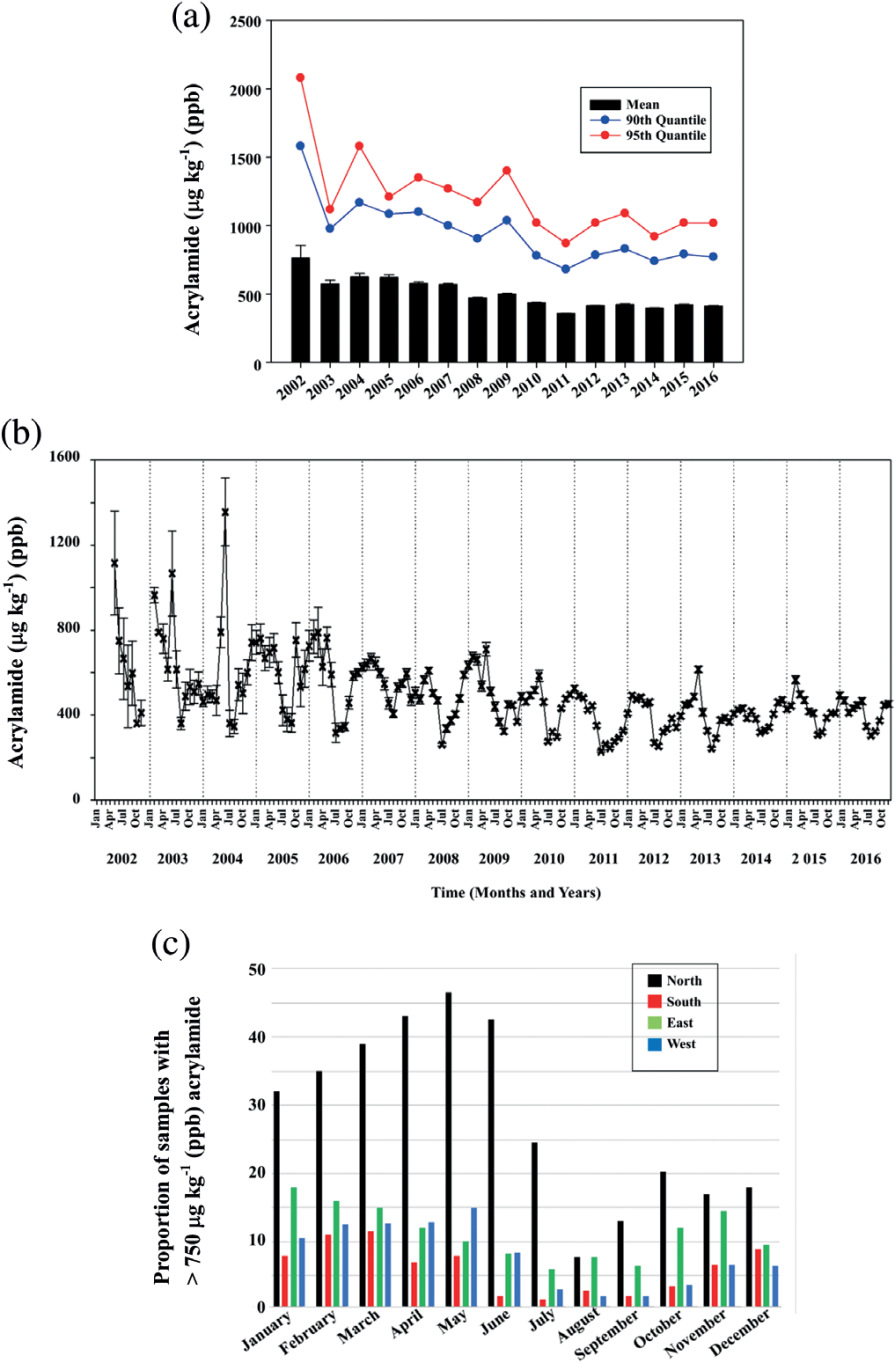
Graphical representations of European manufacturers' data on acrylamide in potato crisps produced from 2002 to 2016. (a) Overall mean acrylamide levels for each year, with standard errors and with trends in 90% and 95% quantiles. (b) Mean acrylamide levels over time (2002–2016) with standard errors, plotted monthly to show seasonality in acrylamide levels. (c) Proportion (%) of samples with more than 750 μg kg^−1^ (ppb) acrylamide for each month over the period 2011–2016 for geographic regions. A and B are reproduced with modification from Powers, Mottram, Curtis, and Halford (2017), while C is plotted using data from the same paper

Whereas the CONTAM Panel report (CONTAM, 2015) expressed concern for the potential neoplastic effects of dietary acrylamide, Regulation (EU) 2017/2158 used stronger language, stating that the Panel's assessment had “confirmed previous evaluations that acrylamide in food potentially increases the risk of developing cancer for consumers in all age groups.” The Regulation replaced Indicative Values with Benchmark Levels, which were lower than the corresponding Indicative Value for almost all product types (Table 1), but described Benchmark Levels as performance indicators rather than triggers for investigation. It is unlikely that consumers or journalists will understand this distinction, and the reduction did not reflect any trend in the data on acrylamide levels in food products. Figure 5a, for example, shows the acrylamide levels in potato crisps produced by manufacturers affiliated to the European Snacks Association, from 2002 to 2016 (Powers et al., 2017; Powers, Mottram, Curtis, & Halford, 2013). The data show a 53% reduction in mean acrylamide levels from 763 μg kg^−1^ in 2002 to 358 μg kg^−1^ in 2011. However, the trend did not continue past 2011, with the mean acrylamide level flattening out and the level in 2016 of 412 μg kg^−1^ being slightly higher than that of 2011. This indicates that the easiest and most effective methods to reduce acrylamide levels had already been implemented by 2011 and further improvements may be difficult to achieve.

Analyses of these data also highlighted two factors that make it more difficult for food producers to achieve regulatory compliance on a consistent basis. The first of these is a seasonal effect (Figure 5b), arising from the fact that European potatoes are harvested between July and October, and for the rest of the year are used from storage. The concentration of reducing sugars is usually the limiting factor for acrylamide formation in potato products, and potatoes in storage are prone to cold and senescent sweetening, both of which bring about an increase in reducing sugar concentrations and are associated with vacuolar invertase (VInv) activity (Clasen et al., 2016; Wiberley‐Bradford & Bethke, 2018; Zhu et al., 2014). Senescent sweetening is also driven by the breakdown of starch through the actions of several enzymes, including phosphorylase L (PhL) and starch‐associated R1 (R1). The second factor is a geographical one, with potatoes grown in northern Europe (Denmark, Finland, Lithuania, Latvia, Norway and Sweden) having a higher acrylamide‐forming potential than potatoes grown elsewhere. The two factors combine to raise the mean acrylamide levels in crisps produced in northern Europe so that the proportion of samples with more than 750 μg kg^−1^ acrylamide (the Benchmark Level set for crisps by the European Commission) is over 30% for the whole of the first half of the year, peaking at more than 45% in May (Figure 5c). Even for the other regions of Europe, the “failure rate” of crisps with respect to the 750 μg kg^−1^ Benchmark Level in some months of the year is over 10%.

This makes it particularly worrying that Regulation (EU) 2017/2158 (European Commission, 2017) includes an explicit threat to impose Maximum Levels for acrylamide in food; that is, levels above which it would be illegal to sell a product. The relevant paragraph of the regulation states that “Complementary to the measures provided for in this Regulation, the setting of Maximum Levels for acrylamide in certain foods should be considered in accordance with Regulation (EEC) No 315/93 following the entry into force of this Regulation”. Regulation 315/93, which came into force in February 1993, states that: “Food containing a contaminant in an amount which is unacceptable from the public health viewpoint and in particular at a toxicological level shall not be placed on the market” (European Commission, 1993). Clearly, if a Maximum Level of 750 ppb were imposed on potato crisps, the industry would not be able to continue as things stand. More detail on the Commission's thinking regarding Maximum Levels for acrylamide was provided when the issue was discussed at a meeting of the European Parliament's Environment, Public Health and Food Safety Committee in January 2017 (European Parliament, 2017). It was stated that the intention was to impose Maximum Levels on sectors of the food industry that do not show “sufficient progress” in reducing acrylamide in their products. Although this is frustratingly vague (what is meant by “sufficient progress”?), we advise anyone in the food production and supply chain to take the threat to impose Maximum Levels seriously.

Regulation (EU) 2017/2158 includes annexes in which mitigation measures to reduce acrylamide formation in different product types are described, from variety selection through crop management to a range of measures that have been shown to be effective during food processing. These are effectively codes of practice, and the wording of the Regulation makes it clear that the adoption of these measures is compulsory: food business “shall” apply the measures that are set out. Ironically for the food industry, many of the measures included in the Regulation are lifted from the FoodDrinkEurope Acrylamide Toolbox (FoodDrinkEurope, 2019) and were originally developed and shared by food businesses. Another key aspect of the Regulation is the requirement for all food businesses to monitor the levels of acrylamide in their products, although businesses that perform retail activities and/or directly supply only local retail establishments are exempt from this requirement. Member States are also required to ensure compliance with the law.

Arguably, the European Union leads the way in developing a regulatory framework for the presence of acrylamide in food. There has been less action in the United States, at least at the Federal level. The Food and Drug Administration (FDA), for example, has not introduced anything equivalent to the EU's Indicative Values or Benchmark Levels, although it has issued an “action plan” with the goals of developing screening methods, assessing dietary exposure and identifying means to reduce it (Food and Drug Administration, 2016). There is, however, an example of a state authority taking action: in 2005, the Attorney General of the State of California filed a lawsuit against five potato crisp and French fry manufacturers (H.J. Heinz, Frito‐Lay, Lance Inc., Kettle Foods and Procter & Gamble) along with four fast‐food chains (McDonald's, Burger King, KFC and Wendy's) for failing to label their products with a Proposition 65 warning to alert consumers to the presence of acrylamide. Proposition 65 is California's “Safe Drinking Water and Toxic Enforcement Act”, which requires businesses to post warnings of any chemical in their products that may cause cancer. The lawsuit was settled in 2008 when the manufacturers committed to cut the level of acrylamide in their products and the fast‐food chains agreed to display acrylamide warnings in their restaurants. Subsequently, in 2010, the Council for Education and Research on Toxics (CERT), a small not‐for‐profit organisation, brought a lawsuit under Proposition 65 against Starbucks and 90 other companies, demanding that the coffee industry remove acrylamide from its products or alert consumers to the presence of acrylamide through warning signs and/or labels. At the time of writing, that case is pending at the Los Angeles Superior Court, but it has prompted the California Office of Environmental Health Hazard Assessment to move towards exempting coffee from Proposition 65 warnings, something that CERT has also raised a legal challenge to.

Other countries in which regulators have taken a position include Canada, where acrylamide has been added to the list of chemicals in the government's Chemicals Management. Health Canada has implemented an acrylamide monitoring program to evaluate the effectiveness of acrylamide reduction strategies it recommends and to assess their implementation by the food industry, with the possibility of setting “reduction targets” in the future. Food Standards Australia New Zealand (FSANZ) and authorities in Japan and Hong Kong have taken similar stances.

## 7 REDUCING THE ACRYLAMIDE‐FORMING POTENTIAL OF POTATOES

Asparagine is the dominant free amino acid in potato tubers, is present typically in concentrations of several 10s of mmol kg^−1^ dry weight, and accounts for approximately one third of the total free amino acid pool. Because it is present in such high concentrations, the concentrations of reducing sugars were initially expected to be the limiting factor for acrylamide‐forming potential, and this was borne out in early studies (Amrein et al., 2003, 2004; Becalski et al., 2004; Burch et al., 2008; de Wilde et al., 2005, 2006). One of those studies, however, did show an effect of free asparagine concentration as well (Becalski et al., 2004), and the relationship between precursor concentration and acrylamide formation in potato has turned out to be quite complex. In 2009–2010, for example, we compared the concentrations of free amino acids and sugars in nine potato varieties supplied by commercial partners. The varieties were classified according to their typical usage, with French fry types Maris Piper, Pentland Dell, King Edward, Daisy and Markies, and crisping types Lady Claire, Lady Rosetta, Saturna and Hermes. The potatoes had been grown in the United Kingdom in 2009 and were analysed at monthly intervals through storage from November 2009 to July 2010.

**Figure 6:**
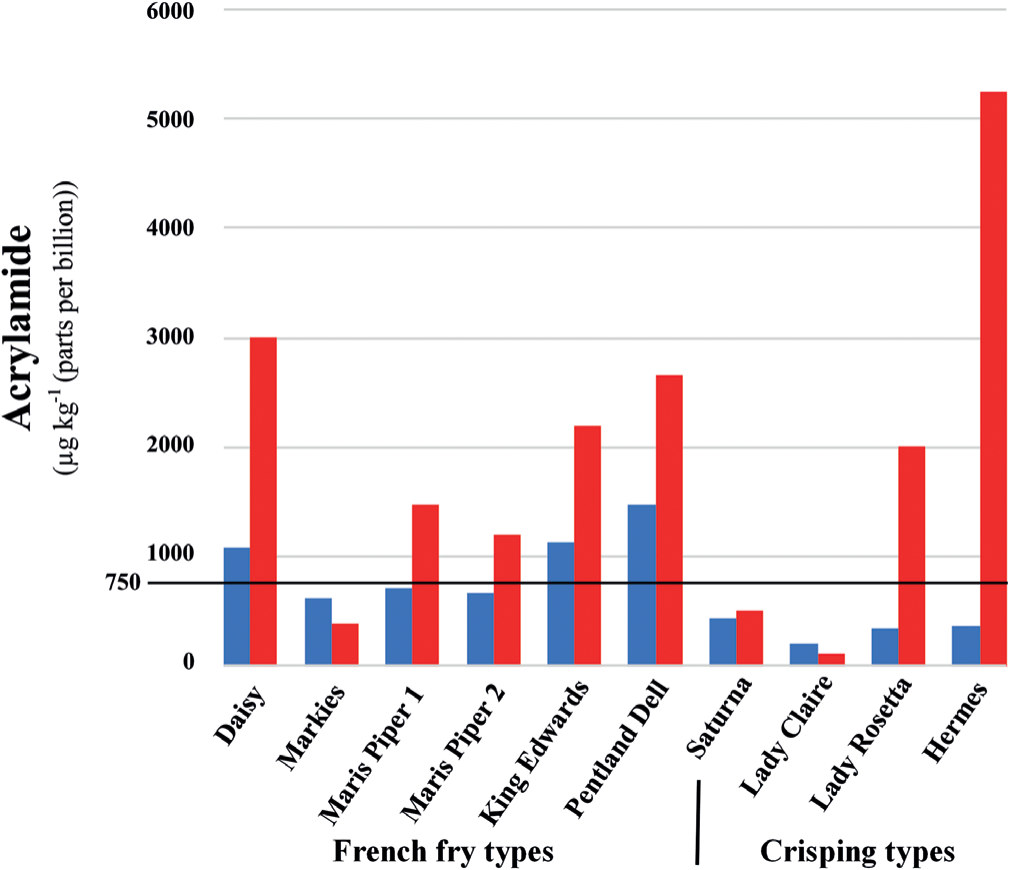
Acrylamide formation in crisps produced from 10 varieties of potato of French fry and crisping types, as indicated. The potatoes were produced by commercial growers in the United Kingdom in 2009 and placed in long‐term storage. The graph shows the results for potatoes sampled in November 2009 (short‐term storage; blue bars) and July 2010 (long‐term storage; red bars). Plotted using data from Halford et al. (2012). The interaction between month (storage time), type and variety factors had a significant (*p* = .039) effect on acrylamide formation (Halford et al., 2012). The current Benchmark Level for acrylamide in potato crisps (750 μg kg^−1^ (ppb)) (European Commission, 2017) is indicated

The acrylamide levels in crisps made from the potatoes in November 2009, when the potatoes had just been placed in storage, and July 2010 at the end of the storage period are shown graphically in Figure 6. The range in acrylamide levels was striking, from approximately 100 μg kg^−1^ for Lady Claire throughout the storage period to over 5,000 μg kg^−1^ for Hermes by the end of the storage period. The varieties also showed contrasting responses to storage, with Lady Rosetta comparable with Lady Claire in November but almost 20 times as high (2013 μg kg^−1^ compared with 104 μg kg^−1^) by the end of the storage period. Markies on the other hand showed the unusual characteristic of decreasing levels of acrylamide formation after long‐term storage.

The study led to the advice that only appropriate potato types and varieties should be used for specific end uses, and that potatoes should be stored at carefully controlled temperatures (typically 8.5–9.5°C) and used within their optimum storage window, which differs between varieties. These measures have been adopted in Regulation (EU) 2017/2158 (European Commission, 2017), although a lower minimum storage temperature of 6°C is stipulated, and are, therefore, now effectively compulsory. Most major food businesses would have been following these practices as a matter of course, anyway, but preventing potatoes from sprouting while storing them at temperatures high enough to avoid cold sweetening is currently dependent on the use of chemical sprout suppressants such as chlorpropham (CIPC; Isopropyl (3‐chlorophenyl)carbamate), and these, too, are coming under the scrutiny of regulators. Indeed, at the time of writing, the European Commission's Standing Committee on Plants, Animals, Food and Feed is considering a proposal for non‐renewal of chlorpropham's authorisation for use as a sprout suppressant in the European Union. This is despite chlorpropham's low level toxicity (Food and Agriculture Organisation of the United Nations, 2001) and is an example of a situation in which the safety implications of banning an agrochemical should be considered together with those of approving it.

Varietal differences and storage were investigated further in a series of field trials in 2010 and 2011 at the Rothamsted Farm site, Woburn, Bedfordshire, UK (Elmore et al., 2015; Muttucumaru et al., 2014, 2017; Muttucumaru, Powers, Elmore, Mottram, & Halford, 2013), and the results of those trials can be summarised as follows. Sucrose was generally the most abundant sugar, as it is in most plant tissues. The exception was Harmony, a boiling variety, in which almost all of the sucrose was converted into glucose and fructose, resulting in very high concentrations of these reducing sugars. French fry varieties generally contained higher concentrations of glucose and fructose than the crisping varieties and showed a trend for an increase in those sugars during storage and for a decline in sucrose concentration. Changes in reducing sugars during storage were variety‐dependent, with some varieties, such as Lady Claire and Verdi, showing consistent stability with respect to reducing sugar content during storage, and Markies again showing a decrease. Most varieties, however, showed an increase in reducing sugar content during storage, and the gradient of this increase steepened as a result of senescent sweetening as the potatoes were kept beyond their optimum storage window, which differed between varieties.

**Figure 7:**
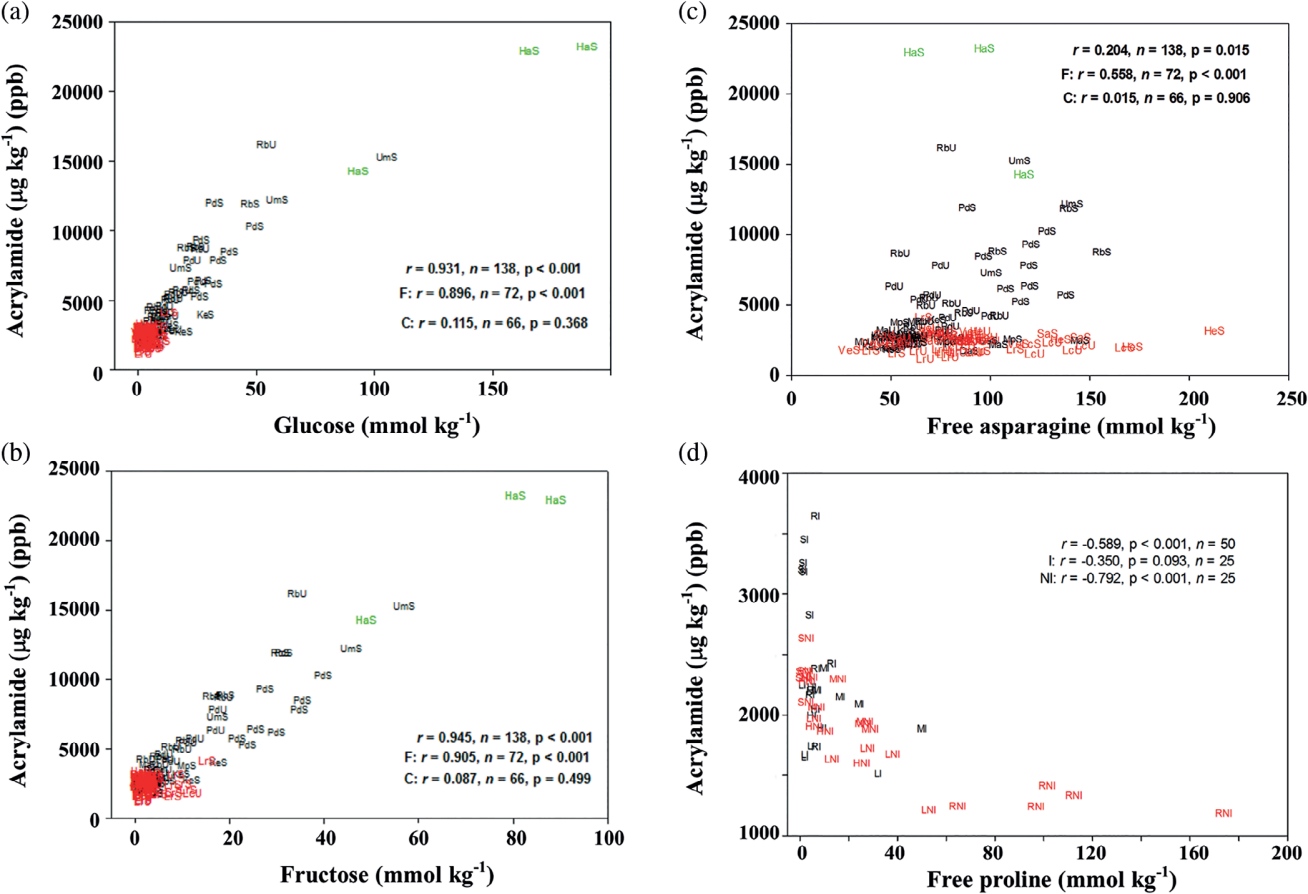
Graphs showing correlations between metabolite concentrations and acrylamide formation in potato flour heated to 160°C for 20 min. (a) Glucose. (b) Fructose. (c) Free asparagine. (d) Free proline. (a–c) Reproduced with modification from Muttucumaru, Powers, et al., 2014, with points on the graphs for French fry varieties shown in black, those for crisping varieties in red and the boiling variety, Harmony, in green. The results for correlation (*r*) are given for all three types overall and then for French fry and crisping types separately. The points are codes for the varieties Maris Piper (Mp), Pentland Dell (Pd), King Edward (Ke), Daisy (D), Markies (Ma), Russet Burbank (Rb), Umatilla Russet (Ur), Lady Claire (Lc), Lady Rosetta (Lr), Saturna (Sa), Hermes (He), Verdi (Ve) and Harmony (Ha), followed by un‐stored (U) or stored (S). (d) Reproduced with modification from Muttucumaru, Powers, Elmore, Mottram, and Halford (2015). Data are represented for five varieties of potatoes grown, with and without irrigation, on commercial farms in the United Kingdom in 2011. The varieties were Hermes (H), Lady Claire (L), Markies (M), Ramos (R), and Saturna (S). Points on the graph from irrigated potatoes are denoted by I in black, while those for not‐irrigated potatoes are denoted by NI in red. The results for correlation (*r*) are given for the whole dataset and separately for irrigated and non‐irrigated potatoes. All concentrations are shown on a dry weight basis

In these studies, acrylamide formation was measured in crisps fried in oil and in flour produced by freeze‐drying tuber material, after the flour had been heated at 160°C for 20 min. The flour method produced more acrylamide than frying crisps, but there was a good correlation between the amounts of acrylamide that formed in the two systems and the flour method is consistent and easier to carry out in a laboratory that is not specialised for food systems. Figure 7a–c shows the relationships between acrylamide formation in heated flour and the concentrations of glucose, fructose and free asparagine in the 2010 field trial (Muttucumaru, Powers, et al., 2014). There were strong correlations between both glucose and fructose concentration and acrylamide formation in the complete dataset. There was also a weak but significant correlation between free asparagine concentration and acrylamide formation in the whole dataset (*r =* .204, *p =* .015) but more strongly in the French fry varieties (*r =* .558, *p <* .001), while there was no significant correlation in the crisping varieties (*r =* .015, *p =* .906). An even stronger effect of free asparagine, not on its own but as a proportion of the free amino acid pool, was observed in potatoes grown under glass (Elmore et al., 2007). However, the wide range in the ratio of free asparagine to other free amino acids that we observed in that pot study has not been reproduced in potatoes from field trials.

A replicate of the 2011 field trial at Woburn was run simultaneously by Higgins Agriculture, approximately 120 miles almost directly north of the Woburn site at Thorn Bank, Doncaster, North Lincolnshire, UK. Comparing the potatoes from the two trials revealed a clear effect of location of cultivation on their acrylamide‐forming potential (Muttucumaru et al., 2017). Most strikingly, free asparagine concentration was much higher in the potatoes grown at the Doncaster site compared with the Woburn site, and free asparagine concentration correlated significantly with acrylamide formation in the Woburn potatoes (*r =* .671, *p <* .001) but not in the Doncaster potatoes (*r =* −.027, *p =* .771). This suggested that the ratio of free asparagine to reducing sugars was an important parameter, and modelling the relationship between this ratio and acrylamide formation calculated that when this ratio is below approximately 2.3:1, free asparagine concentration will contribute to the variance in acrylamide formation, whereas when the ratio is above 2.3:1 reducing sugar concentration alone will determine the formation of acrylamide.

Two other aspects of potato composition are important: the ratio of glucose to fructose and the concentration of free proline. As we have stated already, melanoidin pigments form via similar chemical pathways to acrylamide. Both glucose and fructose can contribute to the formation of colour as well as acrylamide, but fructose has been shown to favour the production of acrylamide over colour during the cooking of French fries, in comparison to glucose (Higley, Kim, Huber, & Smith, 2012; Mestdagh et al., 2008). This suggests that a higher ratio of glucose to fructose could enable a desired product colour to be achieved with less acrylamide formation, consistent with predictions obtained in a study modelling the kinetics of acrylamide formation in French fries (Parker et al., 2012). Data from field trials suggest that the glucose to fructose ratio does not vary greatly (Muttucumaru, Powers, et al., 2014) but any variation that can be found in potatoes or engineered into potatoes genetically could potentially be exploited to reduce acrylamide formation. Meanwhile, blanching French fries to remove the soluble sugars then adding glucose back to enable the Maillard reaction to proceed is included in Regulation (EU) 2017/2158 as an acrylamide mitigation measure.

Free proline has been shown to inhibit acrylamide formation in model systems (Koutsidis et al., 2009), raising the prospect that proline concentration might be another parameter that could be exploited to reduce the acrylamide‐forming potential of potatoes. It is usually present in potatoes at much lower concentrations than free asparagine but has been shown to increase by up to 15‐fold in field‐grown potatoes grown without irrigation, even in the temperate and relatively wet UK (Muttucumaru et al., 2015). The response of potatoes to drought stress is complex: free asparagine concentration, for example, was not affected in the non‐irrigated field‐grown potatoes in that study but almost doubled in response to more severe drought stress imposed in the glasshouse (Muttucumaru et al., 2015). Furthermore, different varieties were affected in dissimilar fashion by the same treatment, indicating that there is no single, unifying potato tuber drought stress response. Nevertheless, the high free proline concentrations in some of the field‐grown potatoes enabled acrylamide formation to be plotted against free proline concentration (Figure 7d), revealing a striking nonlinear relationship (*r* = −.589, *p* < .001) of decreased acrylamide with increased proline.

Lack of irrigation in the field‐grown potatoes also resulted in a lower reducing sugar concentration in four out of five varieties in the study (Lady Claire, Saturna, Ramos and Hermes) and less acrylamide formation in heated potato flour (Muttucumaru et al., 2015). Consequently, the advice to farmers is to irrigate potatoes only if necessary to maintain the health and yield of the crop.

### 7.1 Crop management of potatoes: effects of nitrogen and sulphur fertilisation

The effect of nitrogen fertilisation on the concentration of reducing sugars was investigated long before the acrylamide issue arose because of the implications of reducing sugar concentration for fry colour. In 1990, for example, it was reported that potatoes grown under high nitrogen had lower amounts of free sugar, and consequently tended to have less fry colour, although in high nitrogen potatoes there was more colour per unit of sugar, indicating that amino acids (which were not measured) could be playing a role (Roe, Faulk, & Belsten, 1990). More studies have been conducted since acrylamide was discovered in food, but they have provided conflicting results, probably because the effects of nutrition on potato composition are variety‐specific. Researchers in Belgium, for example, showed that levels of tuber sugars rose in nitrogen‐deprived potatoes by up to 100% compared with adequately fertilised potatoes, resulting in a concomitant increase in acrylamide formation (de Wilde et al., 2006). On the other hand, a Swiss study reported that agricultural practice did not influence the potential for acrylamide formation in potatoes (Amrein et al., 2003).

Our own studies have investigated the effects of sulphur as well as nitrogen nutrition (Muttucumaru et al., 2013). In potatoes grown with different combinations of nitrogen and sulphur in a field trial, glucose concentrations were found to be slightly lower in crisping varieties when nitrogen was supplied at 100 kg per hectare than when it was not supplied, but then increased when nitrogen was applied at 200 kg per hectare. French fry varieties, on the other hand, showed a bigger reduction in glucose concentration from zero to 100 kg per hectare nitrogen, while the concentration at 200 kg per hectare nitrogen was similar to that at zero. There were also different responses of varieties within type, and these type‐ and variety‐specific effects of nitrogen on glucose concentrations probably explain the apparently contradictory results of previous studies. Sulphur, however, had a more consistent effect on glucose concentration, bringing about a reduction of 26% from zero to 40 kg per hectare application.

Nitrogen fertilisation did have a consistent effect on free asparagine concentrations, causing substantial increases in all the varieties. It also caused increases in free glutamine and total free amino acid concentrations, so the suggestion in the 1990 report (Roe et al., 1990) that nitrogen might be affecting amino acid concentrations was correct. Sulphur, however, had no significant effect on free amino acids in the French fry types and an inconsistent effect on the other types.

These complex and confusing changes in free asparagine and reducing sugar concentrations in response to the treatments resulted in nitrogen application having a type‐specific effect on acrylamide‐forming potential, with French fry varieties showing an increase that was not apparent in the crisping varieties. The different varieties within type also showed different responses, with most varieties showing an increase in acrylamide formation under high nitrogen but Pentland Dell and Saturna showing a large decrease, making the situation even more complicated. Sulphur application had no overall effect on acrylamide‐forming potential but did mitigate the effect of high nitrogen application on some of the French fry‐type potatoes, presumably due to its effect on glucose concentrations. We concluded that advice on both nitrogen and sulphur application would have to be carefully tailored for specific varieties, and that there is no quick fix to the acrylamide problem for potatoes through crop management.

## 8 REDUCING THE ACRYLAMIDE‐FORMING POTENTIAL OF WHEAT AND RYE

**Figure 8:**
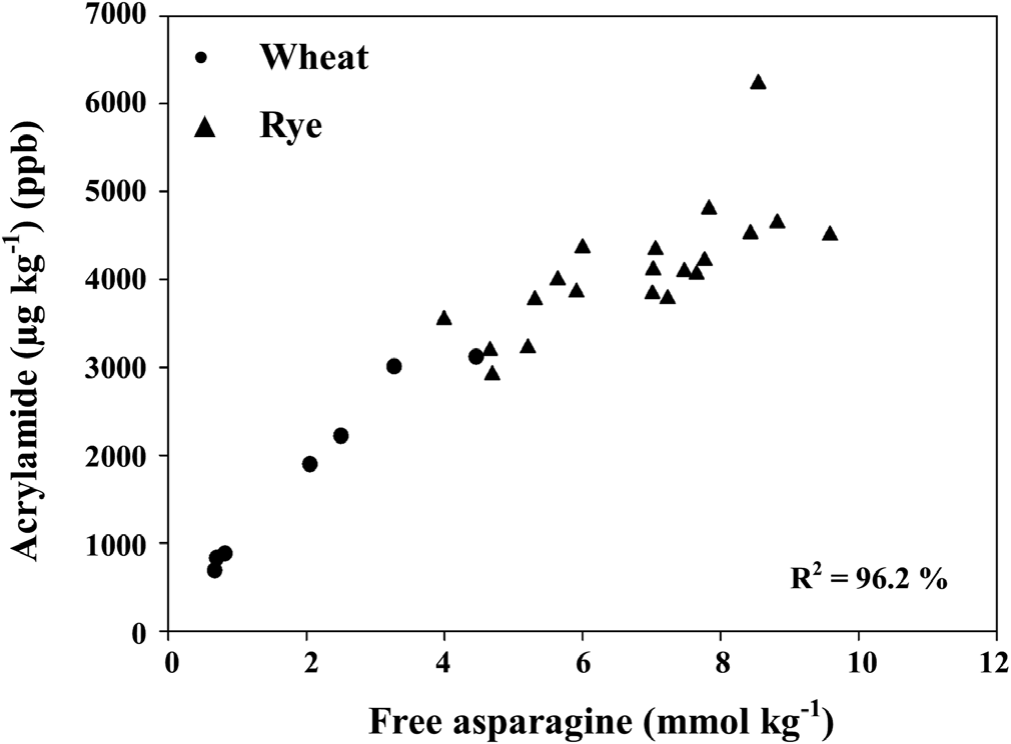
Relationship between free asparagine concentration and acrylamide formation in wheat and rye flour heated for 20 min at 180°C. Reproduced with modification from Curtis, Postles, and Halford (2014)

In cereal grain, in contrast to potato, it is free asparagine concentration that determines the amount of acrylamide that forms during processing (Curtis et al., 2009, 2010; Curtis, Powers, & Halford, 2016; Granvogl et al., 2007; Muttucumaru et al., 2006; Postles, Powers, Elmore, Mottram, & Halford, 2013) (Figure 8). This is probably because there is much less free asparagine in cereal grain, at least from well‐nourished plants, and the ratio of free asparagine to reducing sugars is lower.

### 8.1 Varietal differences in free asparagine concentration

Taeymans et al. (2004) reported large varietal differences in the free asparagine content of European wheat (*Triticum aestivum*) harvested in 2002, while Claus et al. (2006) also reported large differences between varieties, in addition to the effects of agronomic factors. A comparison of six varieties of wheat grown for the UK's Home Grown Cereals Authority (now the Cereals and Oilseeds section of the Agriculture and Horticulture Development Board (AHDB)) at six different sites around the United Kingdom in two harvest years, 2006 and 2007, revealed large differences between varieties, sites and harvest years, and interactions between these factors (Curtis et al., 2009). The bran fractions of milled wheat grain contain more free asparagine than the white flour fraction (Shewry et al., 2009). Analyses of varietal differences have therefore measured free asparagine concentration in wholegrain flour, and this was the case here. Two of the varieties in the study were Claire and Robigus, both of which are classified in the United Kingdom by the National Association of British and Irish Millers (NABIM) as Group 3, soft‐milling wheat varieties, used for biscuits, breakfast cereals, cakes and similar products. The Robigus grain from the different sites and years had a much wider range of free asparagine concentrations (0.67–4.46 mmol kg^−1^) than Claire (0.82–2.68 mmol kg^−1^). Overall, the average for Claire was 1.89 mmol kg^−1^, while the average for Robigus was 2.59 mmol kg^−1^; a difference of 37% with respect to the level in Claire. This study showed that there are substantial varietal differences in free asparagine concentration per se in wheat, and in the response of free asparagine concentration to environmental factors, meaning that varieties need to be tested over a range of environments.

**Figure 9:**
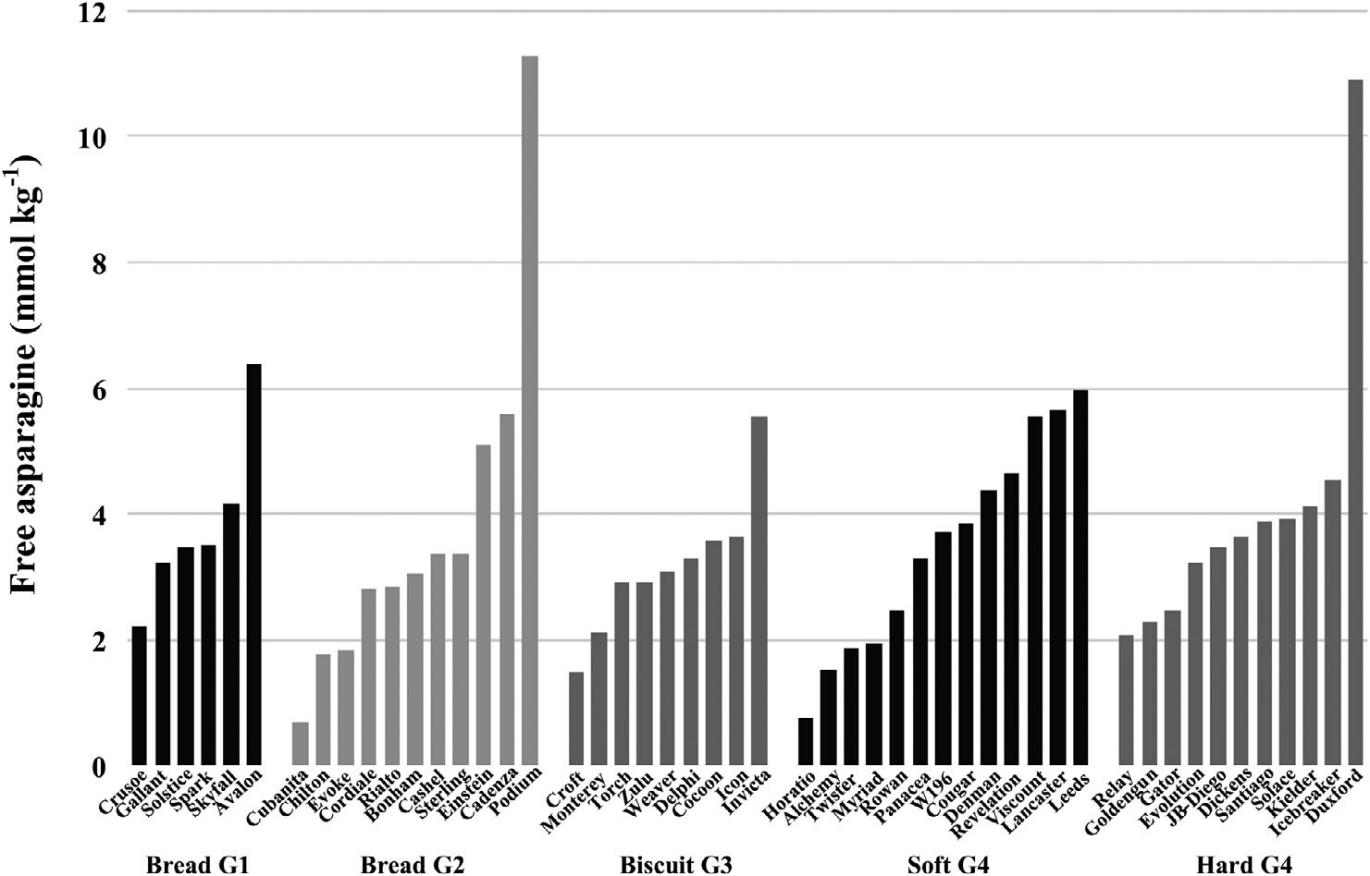
Mean free asparagine concentration in the grain of 50 varieties of winter wheat grown in a field trial in the United Kingdom in 2012–2013. The varieties are grouped according to their classification by the National Association of British and Irish Millers (NABIM), as indicated. There was no significant effect of type (*p* > .05), but a significant effect of variety nested in type (*p* < .001). Replotted from data provided by Curtis, Powers, et al. (2018)

Subsequent field trials of winter wheat varieties confirmed the importance of varietal differences in free asparagine concentration (Curtis, Powers, Wang, & Halford, 2018), and this is illustrated in the plot of data from a 2012 to 2013 field trial in Figure 9. The varieties are grouped according to their classification by NABIM: Group 1, varieties with consistent milling and baking performance; Group 2, varieties with bread‐making potential but not suited to all grists; Group 3, soft varieties used for biscuits, breakfast cereals, cakes and similar products; Group 4, sub‐grouped into hard and soft types; used mainly for animal feed and bioethanol, but incorporated into some grists for food use. The concentration of free asparagine in the grain of these varieties ranged from 0.71 mmol kg^−1^ in Cubanita to 11.29 mmol kg^−1^ in Podium.

While the variety ranking may change from one field trial to another due to environmental factors, several varieties have emerged as being consistently low (Curtis, Powers, et al., 2018). These are Claire, Cocoon, Cordiale, Croft, Delphi, Horatio, Monterey and Myriad. Of these eight varieties, Delphi, Claire, Cocoon, Croft and Monterey are all soft Group 3 biscuit types, while Horatio and Myriad are soft Group 4 types. The eighth variety, Cordiale, is Group 2. However, there was no significant effect of type on free asparagine concentration in, for example, the dataset shown in Figure 9, and selecting grain for processing simply on the basis of it being from a soft‐milling variety would be simplistic and probably ineffective. The free asparagine concentration in the grain from the G3 biscuit wheats in that dataset ranged from 1.48 mmol kg^−1^ in Croft to 5.54 mmol kg^−1^ in Invicta, while in the soft G4 wheats it ranged from 0.76 mmol kg^−1^ in Horatio to 5.99 mmol kg^−1^ in Leeds.

The list of eight varieties with consistently low grain asparagine concentration may be added to in the future because some varieties that have been shown to have low concentrations of free asparagine in one field trial have not been tested over multiple years and sites, so their consistency cannot be assessed. This reflects the annual introduction of multiple new varieties to the United Kingdom wheat seed market, replacing older varieties on the AHDB Recommended List. The problem for food businesses is that by the time there are sufficient data on free asparagine concentration in a variety to make a judgement on it, the variety may already have been dropped from the AHDB Recommended List. We would therefore support free asparagine concentration being measured during variety development.

Despite these problems with varietal comparisons, we strongly advise wheat breeders and farmers to engage on the acrylamide issue. Any modifications that processors have to make to their production lines are expensive and likely to affect product quality. It follows that the cheapest and most straightforward measure that processors could take would be to switch variety, if they could be certain that doing so would reduce the levels of acrylamide in their products, or make the levels more consistent and predictable. There are also anecdotal reports of progress being made in the development of rapid tests for free asparagine concentration that could be applied at the factory gate. This could result in consignments of grain with high free asparagine concentrations being turned away and struggling to find a market other than for animal feed or bioethanol.

**Figure 10:**
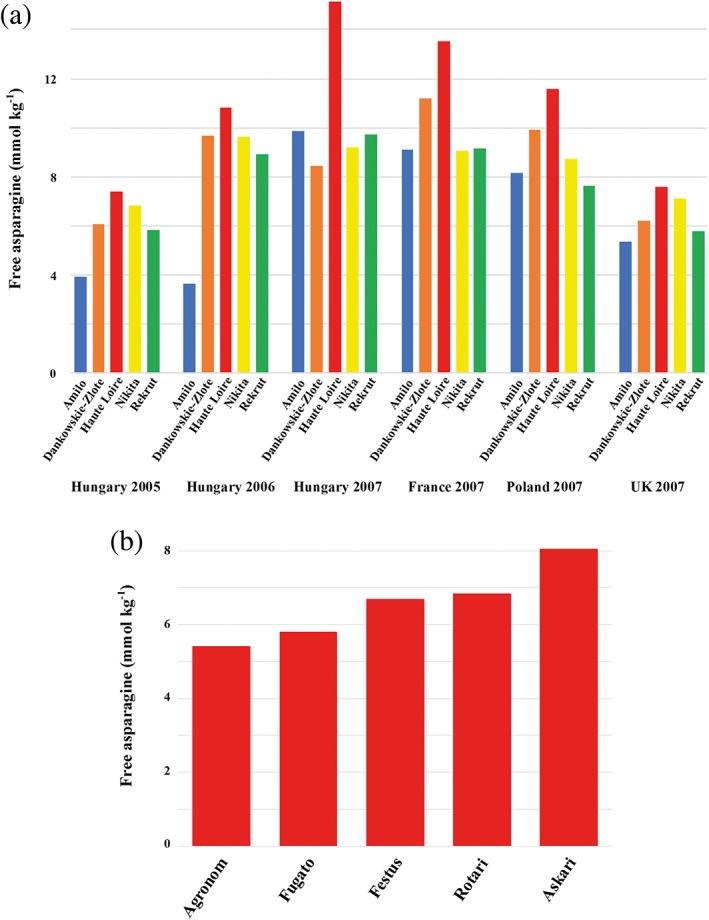
Varietal differences in free asparagine concentration in rye grain. (a) Mean free asparagine concentration in flour from five rye varieties grown in Hungary, Poland, France and the United Kingdom in 2005–2007. There were significant effects (*p* < .05) of country, harvest year and variety. Plotted from data provided by Curtis et al. (2009). (b) Mean free asparagine concentration in grain of five commercially used rye varieties grown in the United Kingdom in 2009–2010. The different varieties accumulated significantly different amounts of free asparagine (*p* = .011). Replotted from data provided by Postles et al. (2013)

Varietal differences in and environmental influences on free asparagine concentration have also been revealed for rye (*Secale cereale*) grain, in an analysis by Curtis et al. (2010). The grain was produced within the European Union Framework 6 (EU FP6) HEALTHGRAIN diversity programme (Ward et al., 2008) at locations in Hungary, France, Poland and the United Kingdom, and harvested in 2005, 2006 and 2007. Free asparagine concentration was closely and positively associated with bran yield, which is particularly important for rye because most rye products are wholegrain. The data for five varieties that were grown in all of the trials are shown graphically in Figure 10a, clearly illustrating the effects of location and harvest year interacting with variety.

A subsequent study analysed the free asparagine concentration in grain from a United Kingdom field trial of five commercial rye varieties (Agronom, Askari, Festus, Fugato and Rotari) and showed considerable differences between the varieties (Figure 10b) (Postles et al., 2013). The difference between the highest asparagine accumulator (Askari) and the lowest (Agronom) was almost 50% with respect to the lower value (8.08 mmol kg^−1^ compared with 5.42 mmol kg^−1^), showing that, as with wheat, processors could potentially reduce the amount of acrylamide forming in their products simply by switching variety.

Another recent study (Žilić et al., 2017) measured free asparagine concentrations not only in bread wheat and rye but also in durum (pasta) wheat (*Triticum durum*), maize (*Zea mays*), barley (*Hordeum vulgare*) and oats (*Avena sativa*). The mean concentration in these species ranged from 8.93 to 3.23 mmol kg^−1^, with the highest mean in rye, followed by oats, maize, durum wheat and bread wheat. There were considerable varietal differences within each species, with the biggest variation in barley (0.91 to 6.26 mmol kg^−1^) and lowest in durum wheat and maize. Wheat varieties from central Europe were also assessed by Rapp, Schwadorf, Leiser, Würschum, and Longin (2018), who reported a relatively tight range of free asparagine concentrations of 1.09 mmol kg^−1^ to 2.98 mmol kg^−1^ in 149 wheat varieties grown at three locations. A wider range, from 2.42 to 11.82 mmol kg^−1^, was measured in 150 wheat genotypes grown for the HEALTHGRAIN diversity programme (Corol et al., 2016). That study also looked at 26 varieties grown over 3 years at four sites across Europe, and while asparagine concentrations were clearly strongly affected by environmental factors across the sites and years it was noted that some genotypes were more consistent than others.

### 8.2 Crop management for low asparagine cereal grains

Asparagine metabolism in plants is influenced by environmental as well as genetic factors (Lea, Sodek, Parry, Shewry, & Halford, 2007). These include nutritional status and pathogen infection, both of which, of course, are directly influenced by crop management. Since free asparagine concentration is the main determinant of acrylamide‐forming potential in cereals, crop management measures are extremely important to reduce the acrylamide‐forming potential of the crop.

As long ago as 1980, increased nitrogen supply was shown to cause a rise in free asparagine concentration in barley grain (Winkler & Schön, 1980), and three years later sulphur was shown to have the opposite effect, with sulphur deficiency bringing about a dramatic increase in free asparagine concentration (Shewry, Franklin, Parmar, Smith, & Miflin, 1983). These effects were investigated further after the discovery of acrylamide in food and the identification of free asparagine as acrylamide's precursor. Increased nitrogen fertilisation of wheat, for example, was reported to lead to increased free asparagine accumulation and more acrylamide formation in bread (Claus, Schreiter, et al., 2006; Martinek et al., 2009), and increases in free asparagine concentration of up to a staggering 30‐fold were measured in the grain of three varieties of wheat, Solstice, Malacca and Claire, in response to severe sulphur deficiency imposed in a pot experiment (Muttucumaru et al., 2006). The concentrations of total free amino acids and free glutamine also rose substantially.

**Figure 11:**
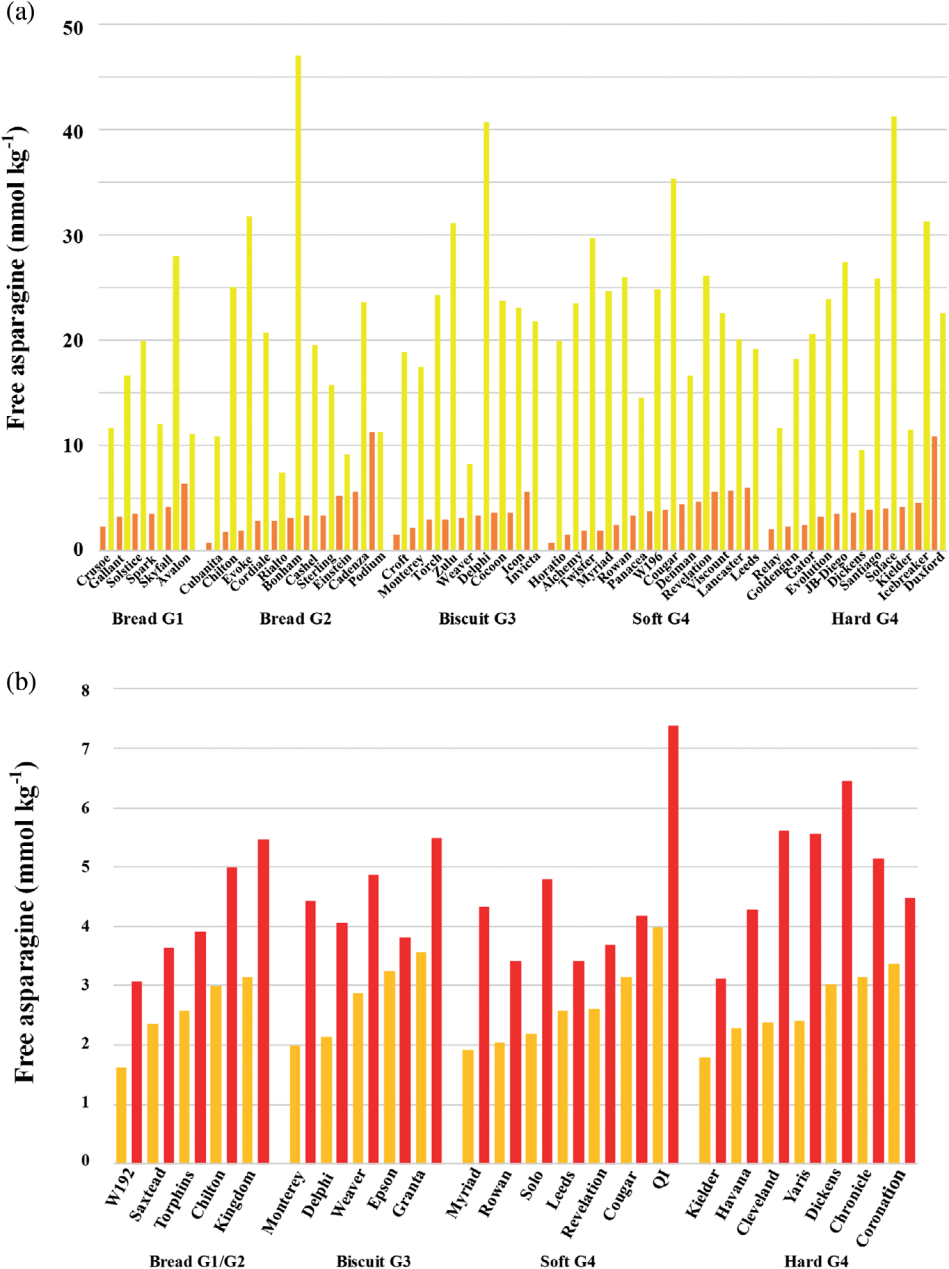
Effects of crop management on free asparagine concentration in wheat. (a) Mean free asparagine concentration in the grain of 50 varieties of winter wheat grown in a field trial in 2012–2013. Data are shown from split‐plots in which wheat in half the plot was supplied with nitrogen and sulphur (brown bars), while the wheat in the other half was supplied with nitrogen but not sulphur (yellow bars). The varieties are grouped according to their classification by the National Association of British and Irish Millers (NABIM), in ascending order for free asparagine concentration in the sulphur supplied condition within each group. There were significant effects of treatment (sulphur supply) (*p* = .007), type interacting with treatment (*p* = .002) and the three‐way interaction of treatment, type and variety nested in type (*p* < .001). Replotted from data provided by Curtis, Powers, et al. (2018). (b) Mean free asparagine concentration in the grain of 24 varieties of wheat grown in a field trial in the United Kingdom in 2011–2012 in which plots were either treated with fungicides (orange) or left untreated (red). There was a strong combined effect of genotype and crop management but this could not be assessed statistically as the untreated plots were not replicated. Replotted from data provided by Curtis et al. (2016)

A series of subsequent studies have confirmed the effect of sulphur deficiency on the free asparagine concentration and acrylamide‐forming potential of wheat grain (Curtis et al., 2009; Curtis, Halford, Powers, McGrath, & Zazzeroni, 2014; Curtis, Powers, et al., 2018; Granvogl et al., 2007). Figure 11a, for example, shows the free asparagine concentration in the grain of 50 winter wheat varieties grown in a field trial in the United Kingdom in 2012–2013 (Curtis, Powers, et al., 2018), with the concentration of sulphur‐fed plants alongside the concentration in sulphur‐deprived plants and the varieties grouped according to their milling type. Analyses of the data showed that different types responded differently to the sulphur treatment, with Group 4 (soft) wheats most affected by sulphur deprivation, but with individual varieties within each type also responding differently to the treatment. Indeed, one variety, Podium, which had one of the highest concentrations of free asparagine in the sulphur‐fed condition, showed no increase at all in the sulphur‐deficient condition (Figure 11a). Overall, varieties with low free asparagine in the sulphur‐fed condition were more affected by sulphur deprivation, meaning that the varietal ranking for free asparagine concentration in the sulphur‐fed condition broke down under conditions of sulphur deficiency.

These studies also provided evidence for the interaction between sulphur feeding and other environmental factors. Two field trials held in the 2011–2012 season in the United Kingdom, for example, showed no effect of sulphur deficiency on free asparagine levels in the grain (Curtis, Halford, et al., 2014; Curtis, Powers, et al., 2018). That season was a highly unusual and difficult one for wheat production in the United Kingdom, in that the crop was poorly established and underwent a dry, cold winter period followed by heavy rainfall in the spring and early summer, then another dry period. However, that season was exceptional, and the advice from the AHDB to United Kingdom farmers is to apply sulphur to wheat at a rate of 20 kg sulphur (50 kg SO_3_ equivalent) per hectare (AHDB, 2014). Ensuring that “good agricultural practices” are followed on fertilisation, to “maintain balanced sulphur levels in the soil and to ensure correct nitrogen application” are also included in Regulation (EU) 2017/2158 (European Commission, 2017).

Nitrogen supply has been shown to have a similar effect on the free asparagine concentration in rye grain to that observed in wheat (Postles et al., 2013). Sulphur application, on the other hand, has no direct effect under field conditions (Postles et al., 2013). Rye may be better able to scavenge available sulphur from the soil than wheat, but may also respond differently to sulphur deficiency. One explanation for the massive accumulation of free asparagine in wheat grain in response to sulphur deficiency is that wheat uses free asparagine as a nitrogen store when it has insufficient sulphur to make sulphur‐rich seed storage proteins (Zhao, Hawkesford, & McGrath, 1999). In contrast, rye appears to accumulate less nitrogen in the grain when it has insufficient sulphur available (Postles et al., 2013).

### 8.3 The importance of disease control

Free asparagine is known to accumulate in many plant species in response to infection by pathogens (Lea et al., 2007), and a study in the Czech Republic suggested that fungicide treatment in wheat could reduce free asparagine accumulation (Martinek et al., 2009). That study involved only three varieties, with an effect observed in trials carried out in 2005–2006 but not 2006–2007. Subsequently, a larger study was carried out in the United Kingdom with grain from 47 commercial United Kingdom varieties grown in a field trial in the 2011–2012 season by Saaten Union (Curtis et al., 2016). The field trial comprised four plots of each variety, three of which were treated with fungicides and the other one not. No disease was recorded above a 5% threshold on the treated plots, while the untreated plot was infected with *Septoria tritici* (also known as *Mycosphaerella graminicola*), with lesser infections of Yellow rust (*Puccinia striiformis*) and Brown rust (*Puccinia triticina*). The severity of the disease in the untreated plot meant that grain could be harvested from only 24 of the 47 varieties.

Figure 11b compares the free asparagine concentrations in grain derived from treated versus untreated plots, with all varieties showing an increase in free asparagine concentration in response to a lack of fungicide treatment. As with the sulphur response (Figure 11a), there were big differences in the degree of change, with free asparagine concentration increasing by only 17%, from 3.3 to 3.8 mmol kg^−1^, in variety Epson, but by 135%, from 2.4 to 5.6 mmol kg^−1^, in Cleveland. Again, as with the effect of sulphur deficiency (Figure 11a), this meant that the ranking of the varieties in the treated plots broke down in the untreated plot (Figure 11b).

While the response of wheat to disease may look superficially similar to the response to sulphur deficiency there were some important differences. For example, while free asparagine became the most abundant free amino acid in the grain in response to sulphur deficiency, the concentration of free aspartic acid increased by more than free asparagine in response to disease, meaning that free aspartic acid, which was the most abundant free amino acid in the fungicide‐treated condition, was also the most abundant in the grain from the untreated plot. Sulphur deficiency also caused an increase in free glutamine concentration, but this did not occur in response to lack of fungicide treatment. This suggests that different mechanisms could be involved in the response to sulphur deficiency and disease, but this requires further investigation.

Free asparagine levels have also been shown to increase in wheat grain in response to treatment with a mycotoxin, deoxynivalenol (DON), produced by another pathogenic fungus, *Fusarium graminearum*, which causes Fusarium head blight (Warth et al., 2015). In that experiment, however, glutamine did increase, as did aspartic acid and glutamic acid. Disease pressure, along with delayed harvest and large kernel size, was also associated with increased free asparagine concentrations in a study of wheat grown in Nebraska (Navrotskyi, Baenziger, Regassa, Guttieri, & Rose, 2018).

The results of these studies show effective disease control to be a second crop management measure for the mitigation of acrylamide formation in wheat products, and this has been adopted in European Commission Regulation (EU) 2017/2158, which states that food businesses must “ensure application of good practices on crop protection measures to prevent fungal infection.” At the same time, however, the European Commission has recently withdrawn or refused to renew the licence for some fungicides, leaving farmers in some cases with no effective products with which to protect their crops. As with chlorpropham, which we discussed in the context of potato storage, this suggests that regulatory authorities are not taking into account all the implications of their decisions on agrochemical use.

### 8.4 Genetic control of free asparagine accumulation in cereal grain

Asparagine synthesis and degradation is a major part of amino acid and nitrogen metabolism in plants. The complexity of its metabolism, including limiting and regulatory factors, has been represented in a detailed network comprising 212 nodes (genes, enzymes or molecules) and 246 edges (reactions between nodes) (Curtis, Bo, Tucker, & Halford, 2018). The core enzymes involved in asparagine metabolism are asparagine synthetase, asparaginase, glutamine synthetase, glutamate dehydrogenase, ferredoxin‐dependent glutamate synthase, NADH‐dependent glutamate synthase, aspartate amino transferase, glutamate decarboxylase and aspartate kinase. Nitrate reductase and nitrite reductase can also be considered. Genes encoding any of these enzymes could be targets for genetic interventions, but to date the focus has been on asparagine synthetase.

Asparagine synthetase catalyses the ATP‐dependent transfer of an amino group from glutamine to aspartate to produce glutamate and asparagine. Asparagine synthetase cDNAs were first isolated by Tsai and Coruzzi (1990) from pea (*Pisum sativum*), and were shown to encode two enzymes, AS1 and AS2. Arabidopsis (*Arabidopsis thaliana*) contains three asparagine synthetase genes, *AtASN1*, *AtASN2* and *AtASN3* (Lam, Hsieh, & Coruzzi, 1998), while potato has two, *StASN1* and *StASN2* (Chawla, Shakya, & Rommens, 2012). Maize, wheat and barley, on the other hand, all have more asparagine synthetase genes, with different genes active in different parts of the plant (Avila‐Ospina, Marmagne, Talbotec, & Krupinska, 2015; Duff et al., 2011; Gao et al., 2016; Todd et al., 2008). Indeed, complexity in the gene family varies considerably between different crop species (Table 2).

**Table 2:** Ensembl database reference numbers for asparagine synthetase genes from selected cereal species

Species	Gene name	Ensembl reference
*T. aestivum*	*TaASN1*	TraesCS5A02G153900, TraesCS5B02G152600, TraesCS5D02G159100
	*TaASN2*	TraesCS3A02G077100, TraesCS3D02G077300
	*TaASN3.1*	TraesCS1A02G382800, TraesCS1B02G408200, TraesCS1D02G390500
	*TaASN3.2*	TraesCS1A02G422100, TraesCS1B02G453600, TraesCS1D02G430300
	*TaASN4*	TraesCS4A02G109900, TraesCS4B02G194400, TraesCS4D02G195100
*T. dicoccoides*	*TdG025640*	TRIDC5AG025640
	*TdG026790*	TRIDC5BG026790
	*TdG009140*	TRIDC3AG009140
	*TdG015200*	TRIDC4AG015200
	*TdG033760*	TRIDC4BG033760
	*TdG056280*	TRIDC1AG056280
	*TdG062090*	TRIDC1AG062090
	*TdG064720*	TRIDC1BG064720
	*TdG071210*	TRIDC1BG071210
*A. tauschii*	*Aet21000200*	AET1Gv21000200
	*Aet20919800*	AET1Gv20919800
	*Aet20170100*	AET3Gv20170100
	*Aet20505300*	AET4Gv20505300
	*Aet20393100*	AET5Gv20393100
*T. urartu*	*Tu05036*	TRIUR3_05036
	*Tu11865*	TRIUR3_27580
	*Tu15772*	TRIUR3_15772
	*Tu21196*	TRIUR3_21196
	*Tu27580*	TRIUR3_27580
*H. vulgare*	*HvASN1*	HORVU5Hr1G048100
	*HvASN2*	HORVU3Hr1G013910
	*HvASN3*	HORVU1Hr1G084370
	*HvASN4*	HORVU1Hr1G092110
	*HvASN5*	HORVU4Hr1G056240
*Z. mays*	*ZmASN1*	Zm00001d045675
	*ZmASN2*	Zm00001d044608
	*ZmASN3*	Zm00001d028750
	*ZmASN4*	Zm00001d047736
	*Zm010355*	Zm00001d010355
	*Zm031563*	Zm00001d031563
	*Zm028766*	Zm00001d028766
*O. sativa*	*OsASN1*	Os03g0291500, BGIOSGA010942
	*OsASN2*	Os06g0265000, BGIOSGA021489

**Figure 12:**
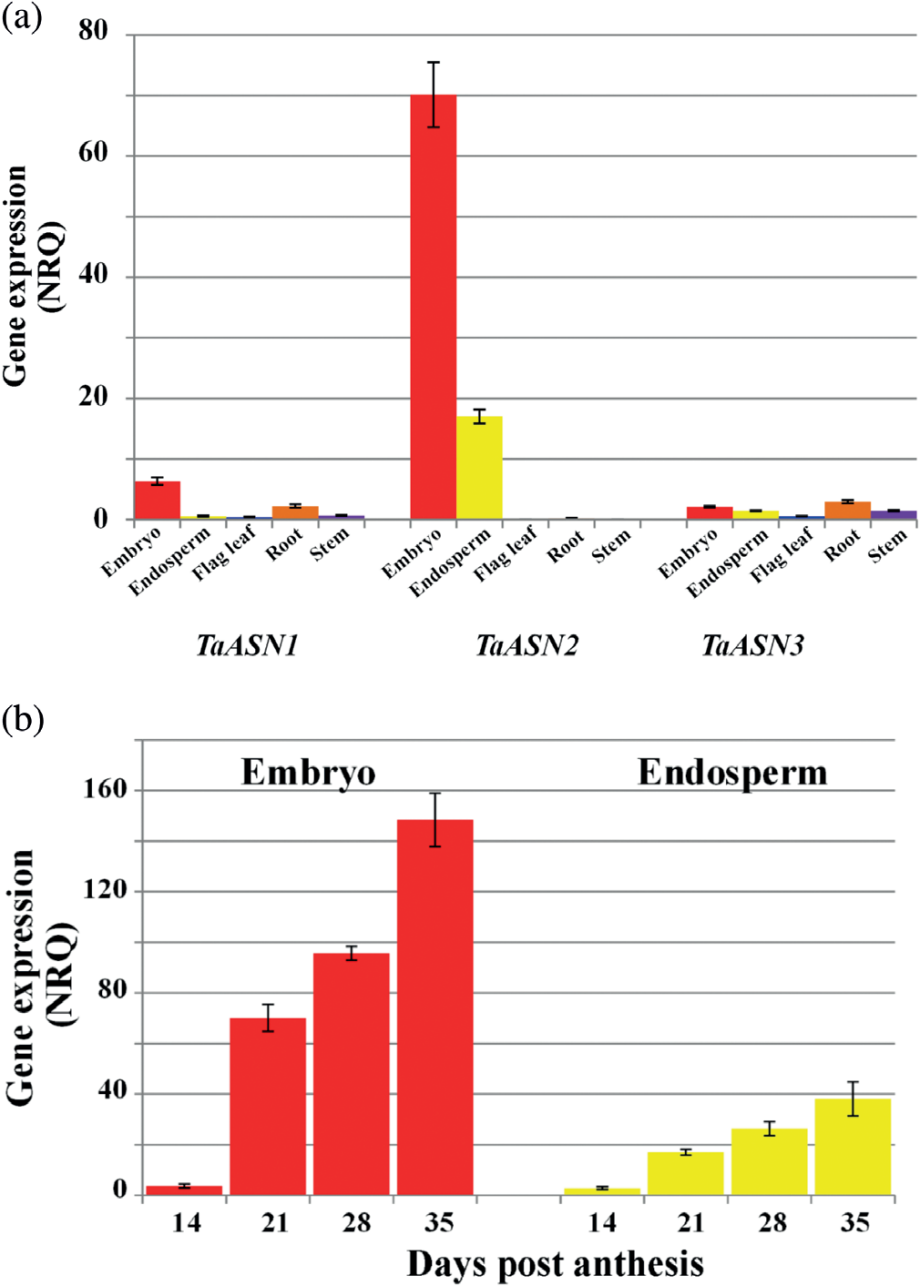
Differential expression of asparagine synthetase genes in wheat. (a) Expression of *TaASN1*, *TaASN2* and *TaASN3* in different tissues of wheat cv. Cadenza plants at 21 days post‐anthesis. (b) Expression of *TaASN1*, *TaASN2* and *TaASN3* in embryo and endosperm tissue of wheat cv. Cadenza plants at 14, 21, 28 and 35 days post‐anthesis. Expression was measured by real‐time PCR and the graphs show the NRQ means and standard errors. Reproduced with modification from Gao et al. (2016)

Of the four asparagine synthetase genes in wheat, *TaASN1*, *TaASN2* and *TaASN4* are present as single copies on Chromosomes 5, 3 and 4, respectively, although some varieties lack a *TaASN2* gene on Chromosome 3B, while there are two copies of *TaASN3* on Chromosome 1 (Xu et al., 2018). *TaASN4* has not been characterised in any detail. Of the others, the expression of *TaASN2* in the embryo and endosperm during mid to late grain development is the highest of any of the genes in any tissue (Figure 12) (Gao et al., 2016), making it a logical target for genetic interventions. However, *TaASN1* is the most responsive to nitrogen availability and sulphur deficiency (Gao et al., 2016) and has also been shown to respond to salt stress, osmotic stress and ABA (Wang, Liu, Sun, & Zhang, 2005).

**Figure 13:**
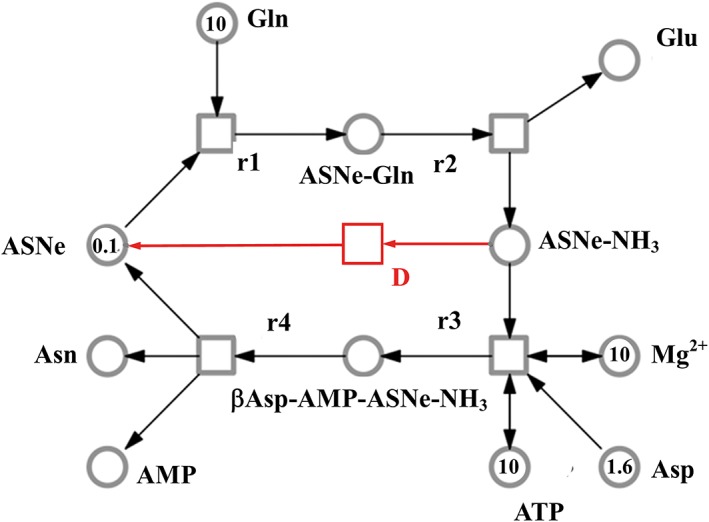
Model representing the reaction catalysed by asparagine synthetase, comprising metabolites (circles), and reactions (squares). The concentration of metabolites is indicated abstractly by numbers in the circles. The model features 11 molecules: AMP, ATP, asparagine (Asn), glutamine (Gln), glutamate (Glu), aspartate (Asp), asparagine synthetase enzyme (ASNe), ASNe complexed with glutamine (ASNe‐Gln), ASNe complexed with ammonia (ASNe‐NH_3_), β‐aspartyl‐complex (βAsp‐AMP‐ASNe‐NH_3_), and magnesium ions. Note that for clarity, water and pyrophosphate are not included. The model was generated assuming that the reactions follow mass action kinetics. The step shown in red represents dissociation of the ASNe‐NH_3_ complex. Reproduced from Xu et al. (2018)

Enzymes TaASN1 and TaASN2 have been expressed heterologously and characterised biochemically (Xu et al., 2018). Both are able to produce asparagine and glutamate from aspartate and glutamine, confirming that they are asparagine synthetases. A continuous Petri net model based on mass‐action kinetics has been constructed using SNOOPY® software to describe the reaction (Xu et al., 2018) (Figure 13). The model comprises 11 molecules (species): adenosine monophosphate (AMP), asparagine (Asn), asparagine synthetase enzyme (for the purpose of the modelling annotated as ASNe), asparagine synthetase enzyme complexed with glutamine (ASNe‐Gln), asparagine synthetase enzyme complexed with ammonia (ASNe‐NH_3_), aspartate (Asp), adenosine triphosphate (ATP), β‐aspartyl‐complex (βAsp‐AMP‐ASNe‐NH_3_), glutamine (Gln), glutamate (Glu) and magnesium ions (Mg^2+^). The experimental data showed that the concentration of glutamate increased at a faster rate than the concentration of asparagine, and the product concentrations plateaued with the concentration of glutamate more than double that of asparagine (Xu et al., 2018). This indicated that the early stages of the reaction (r1 and r2 in Figure 13) could proceed faster than and independently of the later stages (r3 and r4), consistent with the hypothesis proposed by Gaufichon, Reisdorf‐Crena, Rothstein, Chardona, and Suzuki (2010) that steps r1 to r4 occur sequentially rather than simultaneously. So, despite the overall equation of the reaction being Glutamine + Aspartate + ATP → Glutamate + Asparagine + AMP + PPi, glutamate synthesis can proceed independently of asparagine synthesis when aspartate is not available.

Searches of the Ensembl Plants genomic data for *Triticum dicoccoides*, *Aegilops tauschii* and *Triticum urartu*, close relatives of common wheat, reveal the same gene family structure, with four *ASN* genes, three of which are single copy and one of which has two copies (Table 2). This structure is maintained in barley, with a total of four *ASN* genes, one of which is present as two copies on chromosome 1. *HvASN1* (Genbank: AF307145.1) and *HvASN2* (Genbank: AY193714.1), on chromosomes 5 and 3, respectively, were identified and cloned in 2003 (Møller, Taylor, Rasmussen, & Holm, 2003). They were found to be differentially expressed, with only *HvASN1* detected in the roots but both genes expressed in the leaves. This is contrary to wheat, where *TaASN2* expression is believed to be confined solely to the grain (Gao et al., 2016). The other genes were identified by Avila‐Ospina et al. (2015) and annotated as *HvASN3* (Genbank: AK353762), *HvASN4* (Genbank: AK363899) and *HvASN5* (Genbank: AK361923). *HvASN3* and *HvASN4* are both located on chromosome 1 and share 95.93% identity. As such, we would annotate them as *HvASN3.1* and *HvASN3.2*, and *HvASN5* on chromosome 4 as *HvASN4*.

Four *ASN* genes have also been identified in maize (Table 2): *ZmASN1* on chromosome 1, *ZmASN2* on chromosome 3, *ZmASN3* on chromosome 1 and *ZmASN4* on chromosome 9 (Todd et al., 2008). There are also three additional genes that are annotated as *ZmASN1* (Zm010355 on chromosome 8 and Zm031563 and Zm028766 on chromosome 1) but which actually appear to be truncated copies of *ZmASN2*. *ZmASN1* and *ZmASN4* are expressed in all tissues (Todd et al., 2008), with *ZmASN3* expression restricted to below‐ground tissues and *ZmASN2* expression detectable in root, ear node, cob and seed tissues. *ZmASN3* and *ZmASN4* are most similar to *TaASN4*, while *ZmASN2* is most similar to *TaASN1* and *ZmASN2* to *TaASN3*. There is no clear orthologue of *TaASN2*.

In contrast, rice (*Oryza sativa*) only has two *ASN* genes (Table 2). The first (Genbank: D83378) was identified by Watanabe (1996) and further characterised by Nakano, Suzuki, Hayakawa, and Yamaya (2000). The second (Genbank: CI197925.1) was identified through a BLASTN search of the first draft rice genome (Møller et al., 2003) and confirmed by Sakai et al. (2013) and Ohashi et al. (2015), who annotated it as *OsASN1*. *OsASN1* expression was shown to be confined to the roots and to be ammonium‐dependent, while *OsASN2* expression was mainly detected in the leaves but was also found in the roots, where it declined in response to ammonium. Recently, the analysis of mutant lines produced by T‐DNA insertion and CRISPR‐Cas9 has shown *OsASN1* to have a critical role in rice development (Luo et al., 2019). Despite their annotation, *OsASN1* and *OsASN2* are more similar to *TaASN4* and *TaASN3*, respectively, than to *TaASN1* and *TaASN2*. The lack of orthologues of *TaASN1* and *TaASN2* may make rice less prone to accumulating high concentrations of free asparagine in the grain.

In Arabidopsis, *AtASN1*, *AtASN2* and *AtASN3* are expressed in different tissues and differentially regulated by stress stimuli, light and sucrose (Lam et al., 1998). The expression of both *AtASN1* and *AtASN2* is also affected by the supply of organic nitrogen in the form of glutamate, glutamine or asparagine (Lam et al., 1998). However, *AtASN1* expression in tissue culture is repressed by sucrose feeding, whereas *AtASN2* expression is not. The sugar‐sensing signalling pathway in plants involves a protein kinase, sucrose nonfermenting‐1 (SNF1)‐related protein kinase‐1 (SnRK1) (reviewed by Hey, Byrne, & Halford, 2010) and reporter gene expression driven by the Arabidopsis *AtASN1* promoter (also referred to as the dark‐inducible‐6 [*DIN6*] promoter) has been shown to be greatly increased by over‐expression of SnRK1 (Baena‐González, Rolland, Thevelein, & Sheen, 2007; Baena‐González & Sheen, 2008; Confraria, Martinho, Elias, Rubio‐Somoza, & Baena‐González, 2013). The signalling pathway also involves the S1 class of bZIP transcription factors: low sucrose induces asparagine synthetase gene expression via AtbZIP11, while high levels of sucrose induce expression of genes encoding AtbZIP9, AtbZIP10, AtbZIP25 and AtbZIP63, all of which inhibit asparagine synthetase gene expression (Hummel, Rahmani, Smeekens, & Hanson, 2009). It is worth noting, however, that *AtASN1‐3* are not clear orthologues of cereal or other crop asparagine synthetase genes, although *AtASN1* appears similar to wheat *TaASN1*, while *AtASN2* and *AtASN3* may correspond to *TaASN3* and *TaASN4*.

Another protein kinase, general control non‐derepressible‐2 (GCN2), has been shown to affect *TaASN1* gene expression in wheat (Byrne et al., 2012). GCN2 phosphorylates the α subunit of translation initiation factor eIF2 (eIF2α). In fungi, it is activated in response to a reduction in free amino acid concentrations and maintains the balance between free amino acids and proteins (reviewed by Hinnebusch, 1992). An Arabidopsis homologue has been shown to be activated in response to herbicides that inhibit amino acid biosynthesis (Zhang et al., 2008; Zhang, Dickinson, Paul, & Halford, 2003), as well as multiple stress stimuli, including purine deprivation, UV light, cold shock, wounding, pathogen infection, methyl jasmonate, salicylic acid and cadmium exposure (Lageix et al., 2008). Over‐expression of the wheat homologue, *TaGCN2*, in transgenic wheat resulted in significant decreases in total free amino acid concentration in the grain, with free asparagine concentration in particular being much lower than in controls, and reduced expression of *TaASN1* (Byrne et al., 2012). Sulphur deficiency‐induced activation of *TaASN1* occurred in wild‐type plants but not in *TaGCN2* over‐expressing lines (Byrne et al., 2012). GCN2 activity has also been linked with asparagine synthetase gene expression and sulphur metabolism in mammalian systems: phosphorylation of eIF2α and expression of asparagine synthetase genes have both been shown to be higher in liver cells of rats fed a diet deficient in sulphur‐containing amino acids than of well‐nourished rats (Sikalidis & Stipanuk, 2010).

**Figure 14:**
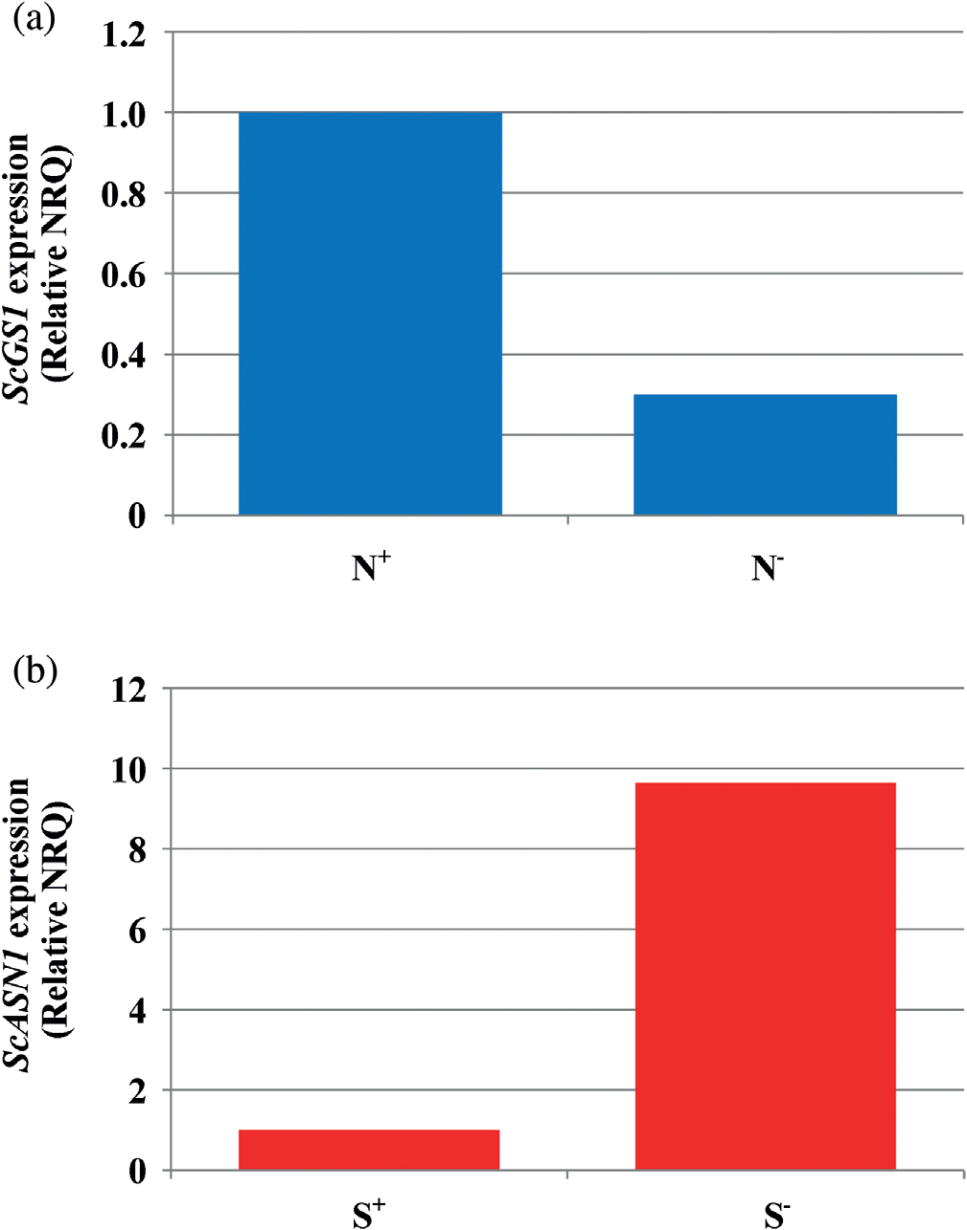
Gene expression in rye (cv. Askari) grain at 21 days post anthesis. (a) Expression of glutamine synthetase‐1 gene (*ScGS1*) in grain produced under different nitrogen treatments (shown as N+ and N−). The effect of nitrogen was significant (*p* < .05). (b) Expression of asparagine synthetase‐1 gene (*ScASN1*) in grain produced under different sulphur treatments (shown as S+ and S−). The effect of sulphur was marginally significant (*p* = .089). Mean normalised relative quantities (NRQ) are shown with the NRQ for the control treatment (N+ in A and S+ in B) set at 1

Complete genome data is not yet available for rye, but homologues of several genes involved in asparagine metabolism have been identified and characterised (Postles et al., 2016). These are asparagine synthetase‐1 (*ScASN1*), glutamine synthetase‐1 (*ScGS1*), potassium‐dependent asparaginase (*ScASP*), aspartate kinase (*ScASK*), and general control non‐derepressible‐2 (*ScGCN2*). *ScGS1* expression in the grain at 21 days post‐anthesis was shown to increase in response to higher nitrogen availability (Figure 14a). There was also a large but only marginally significant (*F* test, .05 < *p* < .10) increase in *ScASN1* gene expression in response to sulphur deficiency (Figure 14b).

## 9 PROSPECTS FOR REDUCING ACRYLAMIDE‐FORMING POTENTIAL BY PLANT BREEDING

An important question for breeders is how much could breeding reduce the acrylamide‐forming potential of crops. For cereals that means reducing the free asparagine concentration in the grain. Breeders may have been discouraged from including this trait as a breeding target because of the response of free asparagine concentration to environmental and crop management factors. That attitude has changed as pressure from food businesses for low asparagine varieties has increased.

If breeders are to make progress they will need genetic resources in the form of quantitative trait loci and genetic markers. The development of such resources has been slow, but Rapp et al. (2018) have reported the identification of some QTL using genome‐wide association mapping. The strongest QTL only accounted for 18% of the genotypic variance in free asparagine concentration, but the study also found moderately high heritability of the trait (.65) and achieved a respectable cross‐validated prediction ability of .62 by combining the QTL with a genome‐wide prediction approach. Importantly, the trait showed no correlations with protein content, sedimentation volume or falling number. The authors' recommendation for dealing with environmentally induced variation in free asparagine concentration was to use marker‐assisted or genomic selection in early generations of a breeding programme and only test for free asparagine concentration in later generations.

In potato (*Solanum tuberosum*), the predominant role of reducing sugar concentration in determining the acrylamide‐forming potential of potatoes (Figure 7) makes genes encoding enzymes involved in the production, accumulation and turnover of these reducing sugars, and the stability of sugar concentrations during storage, obvious potential targets for genetic interventions. However, crisping varieties in particular have been bred for low glucose and fructose concentrations for many years and the lowest may already be close to the minimum to be “fryable.” In addition, the concentration of reducing sugars affects all products of the Maillard reaction, including flavour, colour and aroma compounds, potentially impacting on product quality. Interventions to increase the ratio of glucose to fructose may get around this problem by favouring the formation of colour and possibly other desirable reaction products over acrylamide, but, as we have stated already, there appears to be little variation in that parameter in the varieties that have been analysed so far. Low free asparagine concentration, both per se and as a proportion of the total free amino acid pool, may, therefore, be a better target. Our advice to breeders at present is to pursue all approaches until we know more about the outcomes, positive and negative.

A potato population segregating for reducing sugar and asparagine content has been used to identify putative QTL for free asparagine and reducing sugars (Shepherd et al., 2010, 2013). QTL have also been identified for fry colour (Bradshaw, Hackett, Pande, Waugh, & Bryan, 2008) and these are likely to relate to reducing sugar concentrations. Comparative transcriptomic analysis of two genotypes from the population showed expression of an asparagine synthetase gene to be highly positively associated with asparagine content (Shepherd et al., 2013).

## 10 BIOTECH APPROACHES TO REDUCING THE ACRYLAMIDE‐FORMING POTENTIAL OF CROPS

Low acrylamide GM potato varieties, called Innate® and Innate® Generation 2, are already being marketed in the USA and Canada by the Simplot Company of Boise, Idaho. The company initially targeted asparagine synthetase: potato has two asparagine synthetase genes, *StASN1* and *StASN2*, and expression of both was reduced in a popular American French fry variety, Ranger Russet, using RNA interference (Rommens, Yan, Swords, Richael, & Ye, 2008). This resulted in a 95% reduction in free asparagine concentration in the tubers, but the plants produced small, cracked tubers when grown in the field. However, plants in which *StASN1* expression alone was targeted in a tuber‐specific manner did not have this problem (Chawla et al., 2012), producing normal yields of potatoes with very low concentrations of free asparagine. This trait has been incorporated into both Innate® and Innate® Generation 2 (USDA‐APHIS, 2013, 2014). The effectiveness of reducing the expression of *StASN1* specifically in the tubers is consistent with the free asparagine that accumulates in the tubers being synthesised in situ rather than imported from elsewhere in the plant (Karley, Douglas, & Parker, 2002; Muttucumaru, Keys, Parry, Powers, & Halford, 2014).

Innate® and Innate® Generation 2 also have reduced expression of genes *PhL* (starch phosphorylase L) and *R1* (starch‐associated R1), resulting in less starch breakdown during storage, as well as reduced bruising through reduced expression of gene *PPO5* (polyphenol oxidase) (USDA‐APHIS, 2013, 2014). Innate® Generation 2 also has reduced expression of a vacuolar invertase gene (*VInv*), so is less prone to cold and senescent sweetening and the associated increases in glucose and fructose concentration (USDA‐APHIS, 2014). In addition, it has increased resistance to late blight disease (*Phytophthora infestans*) through incorporation of a resistance gene, *Rpi‐vnt1.1*, from a wild potato species, *Solanum venturii* (USDA‐APHIS, 2014). The low concentrations of free asparagine and reducing sugars in the tubers of Innate® Generation 2 are claimed to reduce acrylamide‐forming potential by 90% compared with conventional potatoes. A reduction of 90% in acrylamide levels would ensure regulatory compliance for European French fry and crisp manufacturers, at least in the context of current Benchmark Levels. Unfortunately, there is currently no prospect of these or any other GM varieties being grown in Europe, highlighting how far behind crop biotechnology is in Europe compared with the USA, and how this is beginning to compromise efforts to improve food safety.

The vacuolar invertase (*VInv*) gene has also been targeted using a gene editing technique. TALENs (transcription activator‐like effector nuclease) in French fry variety, Ranger Russet (Clasen et al., 2016). Tubers from lines with a full knockout of the gene had undetectable levels of reducing sugars, and crisps produced from them had much lower levels of acrylamide than crisps produced from control tubers. Varieties carrying this trait have not been commercialised yet. It is possible that a genome editing technique was used in this case because there is no prospect at all of GM varieties being developed for the European market. However, at the time of writing, prospects for the commercialisation of genome edited crop varieties in Europe also look bleak (Halford, 2019).

## 11 CONCLUDING REMARKS

The presence of acrylamide in many popular foods has become one of the most difficult problems facing the food industry. Consumers have been remarkably unmoved by the issue, but regulators have not, and while that situation lasts the problem is principally one of regulatory compliance. That does not, necessarily, make it any easier for the food industry to deal with, and the difficulty of reducing the levels of acrylamide in food products is compounded by the fact that many of the compounds that impart colour, flavour and aroma to a product, giving it the characteristics that are demanded by consumers and define food types and brands, form by similar pathways to acrylamide. Nevertheless, the direction of travel of regulations in the European Union is unwaveringly towards Maximum Levels, that is, the setting of levels of acrylamide above which it would be illegal to sell a food product, and other regulatory authorities around the world may follow the European Union's lead. The imposition of Maximum Levels at the current Benchmark Levels set in the European Union would be extremely difficult for the food industry to cope with and we urge all stakeholders in the food supply chain to engage on the acrylamide issue in order to enable food businesses to stay ahead of regulatory developments.

Lowering the levels of acrylamide in food may be achieved by modifying processes and/or reducing the acrylamide‐forming potential of the raw material. We have not reviewed work on the first of these in detail, but the methods that have been shown to work have been distilled in the FoodDrinkEurope Toolbox (FoodDrinkEurope, 2019) and have been reviewed comprehensively by Friedman (2015). Instead, we have focused on genetic and agronomic approaches to addressing the acrylamide problem, which, if successful, could eventually lead to huge savings for the food industry through the avoidance of costly modifications to manufacturing lines or the loss of valuable product types and brands, dwarfing the cost of the research involved. Indeed, the development of modern techniques for genetic interventions, including chemical mutagenesis coupled with genomics, genetic modification and genome editing, make step reductions in the acrylamide‐forming potential of potatoes and cereals a realistic possibility.

We conclude by quoting the most commonly used definition of food security, which comes from the Food and Agriculture Organisation of the United Nations (2003): “Food security exists when all people, at all times, have physical, social and economic access to sufficient, safe and nutritious food to meet their dietary needs and food preferences for an active and healthy life.” In other words, there can be no food security without food safety.
